# Activation of HIV Transcription with Short-Course Vorinostat in HIV-Infected Patients on Suppressive Antiretroviral Therapy

**DOI:** 10.1371/journal.ppat.1004473

**Published:** 2014-11-13

**Authors:** Julian H. Elliott, Fiona Wightman, Ajantha Solomon, Khader Ghneim, Jeffrey Ahlers, Mark J. Cameron, Miranda Z. Smith, Tim Spelman, James McMahon, Pushparaj Velayudham, Gregor Brown, Janine Roney, Jo Watson, Miles H. Prince, Jennifer F. Hoy, Nicolas Chomont, Rémi Fromentin, Francesco A. Procopio, Joumana Zeidan, Sarah Palmer, Lina Odevall, Ricky W. Johnstone, Ben P. Martin, Elizabeth Sinclair, Steven G. Deeks, Daria J. Hazuda, Paul U. Cameron, Rafick-Pierre Sékaly, Sharon R. Lewin

**Affiliations:** 1 Department of Infectious Diseases, Alfred Hospital and Monash University, Melbourne, Victoria, Australia; 2 Centre for Biomedical Research, Burnet Institute, Melbourne, Victoria, Australia; 3 Doherty Institute for Infection and Immunity, University of Melbourne, Melbourne, Victoria, Australia; 4 Vaccine Gene Therapy Institute Florida, Port St Lucie, Florida, United States of America; 5 National Association of People Living with HIV Australia, Sydney, New South Wales, Australia; 6 Peter MacCallum Cancer Institute, Melbourne, Victoria, Australia; 7 Peter MacCallum Department of Oncology, University of Melbourne, Melbourne, Victoria, Australia; 8 Westmead Millenium Institute, University of Sydney, Westmead, New South Wales, Australia; 9 Karolinska Institutet, Stockholm, Sweden; 10 University of California San Francisco, San Francisco, California, United States of America; 11 Merck Research Laboratories, West Point, Pennsylvania, United States of America; Duke University Medical Center, United States of America

## Abstract

Human immunodeficiency virus (HIV) persistence in latently infected resting memory CD4+ T-cells is the major barrier to HIV cure. Cellular histone deacetylases (HDACs) are important in maintaining HIV latency and histone deacetylase inhibitors (HDACi) may reverse latency by activating HIV transcription from latently infected CD4+ T-cells. We performed a single arm, open label, proof-of-concept study in which vorinostat, a pan-HDACi, was administered 400 mg orally once daily for 14 days to 20 HIV-infected individuals on suppressive antiretroviral therapy (ART). The primary endpoint was change in cell associated unspliced (CA-US) HIV RNA in total CD4+ T-cells from blood at day 14. The study is registered at ClinicalTrials.gov (NCT01365065). Vorinostat was safe and well tolerated and there were no dose modifications or study drug discontinuations. CA-US HIV RNA in blood increased significantly in 18/20 patients (90%) with a median fold change from baseline to peak value of 7.4 (IQR 3.4, 9.1). CA-US RNA was significantly elevated 8 hours post drug and remained elevated 70 days after last dose. Significant early changes in expression of genes associated with chromatin remodeling and activation of HIV transcription correlated with the magnitude of increased CA-US HIV RNA. There were no statistically significant changes in plasma HIV RNA, concentration of HIV DNA, integrated DNA, inducible virus in CD4+ T-cells or markers of T-cell activation. Vorinostat induced a significant and sustained increase in HIV transcription from latency in the majority of HIV-infected patients. However, additional interventions will be needed to efficiently induce virus production and ultimately eliminate latently infected cells.

**Trial Registration:**

ClinicalTrials.gov NCT01365065

## Author Summary

The major barrier to curing HIV is the long term persistence of latently infected resting memory T-cells in HIV-infected patients on antiretroviral therapy (ART). One strategy being pursued to eliminate latently infected cells is to activate HIV production from latently infected cells with the aim of killing latently infected cells via virus induced cell death or stimulation of an HIV-specific immune response. Histone deacetylases (HDACs) are important in maintaining HIV latency. Vorinostat, an inhibitor of HDACs (HDACi) licensed for the treatment of some malignancies, has been shown in laboratory studies and a clinical study of selected individuals to disrupt HIV latency. We examined the ability of standard dose vorinostat given daily for 14 days to activate latent HIV infection in unselected HIV-infected individuals on ART. The study showed evidence of activation of latent HIV infection in 18/20 (90%) of individuals and was safe and generally well tolerated. There were significant early changes in host gene expression, which persisted during and after the period of vorinostat. No changes were seen in immune activation or number of latently infected cells. Vorinostat was able to activate latent HIV infection in most individuals. Additional interventions will be needed to eliminate latent HIV infection.

## Introduction

One of the major barriers to a cure for human immunodeficiency virus (HIV) infection are long lived latently infected memory CD4+ T-cells that persist in patients on suppressive antiretroviral therapy (ART) [1], [2]. One approach currently being investigated to eliminate latently infected cells is to induce production of virus from latently infected cells making the recently activated latently infected cell susceptible to death from virus-induced cytolysis or induction of HIV-specific T-cells [3].

Histone deacetylase inhibitors (HDACi) can activate HIV production efficiently in nearly all latently infected cell lines [4]–[9] and in many but not all primary CD4^+^ T-cell models of latency [10]. Using resting CD4^+^ T-cells from HIV-infected patients on cART *ex vivo*, HDACi induce both virus transcription and production of free virus [11], although the amount of virus produced from resting CD4+ T-cells is significantly less than that induced by a T-cell mitogen [12]. The pan HDACi vorinostat, the first HDACi to be licensed for the treatment of cutaneous T-cell lymphoma [13] is a less potent activator of latent HIV than other HDACi such as romidepsin [11], [12], [14], but has been clearly shown to induce virus production from resting CD4^+^ T-cells from HIV-infected patients on cART *ex vivo* by some groups in both the absence [15] or presence [8], [16] of activated feeder cells while other groups have shown minimal virus production [12], [17]. There has been recent data suggesting that HDACi may only activate HIV transcription through stimulation of a host gene promoter leading to the production of chimeric host-HIV transcripts, or read-through transcripts, and not true CA-US HIV RNA raising a concern that HDACi are unable to induce virion production [12]. However, studies using other models of ex vivo stimulation of resting CD4+ T-cells from HIV-infected patients on ART have not supported these findings [11]. Furthermore, recent data from a clinical trial of the HDACi romidepsin clearly demonstrated that virus could be produced in vivo following intravenous administration of this HDACi to HIV-infected patients on ART [18].

Recently, vorinostat was demonstrated to activate HIV transcription *in vivo* in resting memory CD4+ T-cells in HIV-infected subjects on ART who had been selected based upon *ex vivo* increase in HIV transcription by vorinostat [16], [19]. We hypothesized that a multi-dose course of vorinostat would increase HIV transcription in CD4+ T-cells in blood from unselected HIV-infected patients on suppressive ART. We aimed to determine the safety and tolerability of short course vorinostat in HIV-infected patients on ART and to determine the effect on cell associated unspliced (CA-US) HIV RNA in CD4+ T-cells and the number of latently infected cells in blood and rectal tissue.

## Results

### Multiple doses of vorinostat were well tolerated and induced an increase in histone acetylation and cell associated unspliced HIV RNA

Participants' median baseline CD4+ T-cell count was 721 (IQR 621, 907) cells/µl and duration of virus suppression was 5.0 (IQR 3.9, 7.5) years (**Table 1**). All enrolled subjects completed the study as planned. Adverse events were mild or moderate in severity (**Tables S1 and S2**) and there were no significant interactions with ART (**Table S3**).

**Table 1:** Baseline characteristics of study participants.

Patient ID	Gender (M = Male, F = Female)	Age (years)	Baseline CD4 (cells/µL)	Duration of virological suppression (years)	Regimen^c^	NNRTI or PI based regimen
VOR001	M	49.8	710	5.0^b^	TDF+3TC+EFV once daily	NNRTI
VOR002	M	51.2	494	7.7	TDF/FTC once daily+NVP twice daily	NNRTI
VOR003	M	56.6	479	4.0^b^	TDF/FTC/EFV once daily	NNRTI
VOR004	M	40.8	725	13.4	ABC/3TC once daily+NVP twice daily	NNRTI
VOR006	F	41.0	743	11.0	TDF/FTC/EFV once daily	NNRTI
VOR008	M	49.2	863	5.6	TDF/FTC/EFV once daily	NNRTI
VOR009	M	54.9	703	7.5	TDF once daily+3TC once daily+NVP twice daily	NNRTI
VOR010	M	49.3	371	4.4	TDF/FTC once daily+NVP twice daily	NNRTI
VOR011	M	55.6	1136	7.4	TDF/FTC/EFV once daily	NNRTI
VOR013	M	45.0	1098	5.9^b^	TDF/FTC+DRV+r once daily	PI
VOR014	M	43.6	717	3.5	TDF/FTC/EFV once daily	NNRTI
VOR015	M	40.4	538	3.7	TDF/FTC/EFV once daily	NNRTI
VOR016	M	43.2	951	4.9^b^	AZT+TDF+LPV+r twice daily	PI
VOR017	M	47.5	1335	4.5^b^	ABC/3TC once daily+NVP once daily	NNRTI
VOR018	M	48.4	561	2.7	ABC/3TC+ATV+r once daily	PI
VOR019	M	42.9	855	3.5	TDF/FTC/EFV once daily	NNRTI
VOR020	M	45.4	694	4.0^b^	ABC/3TC once daily+TDF once daily+DRV+r twice daily+RAL twice daily	PI/INI
VOR021	M	35.0	658	3.6	TDF/FTC+ATV+r once daily	PI
VOR022	M	52.7	1094	7.9	AZT+3TC+LPV+r twice daily	PI
VOR023	M	53.7	727	7.5	TDF/FTC+NVP SR once daily	NNRTI
**Summary Value** a	**19 Male (95%)**	**47.9 (43.1–52.0)**	**721 (610–907)**	**5.0 (3.9–7.5)**		

a Values represent n (% with characteristic) or median (interquartile range).

b Patient had a single HIV RNA viral load to <200 copies/mL during period of suppression.

c Antiretroviral agents separated by “/” were fixed dose combination.

“+r” denotes administration of ritonavir.

NOTE: TDF, Tenofovir; 3TC, Lamivudine; FTC, Emtricitabine; EFV, Efavirenz; NVP, Nevirapine; ABC, Abacavir; 3TC, Lamivudine; DRV, Darunavir; RTV, Ritonavir; AZT, Zidovudine; LPV, Lopinavir; RAL, Raltegravir; ATV, Atazanavir; NVP SR Nevirapine (slow release).

**Figure 1:**
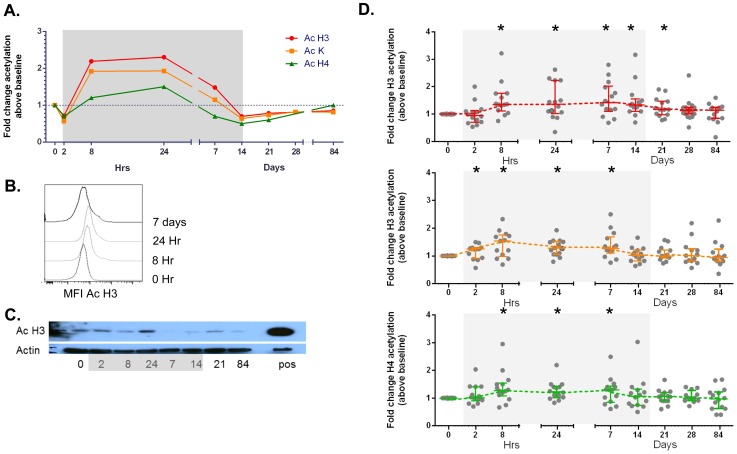
Induction of changes in acetylation of histone 3, histone 4 and lysine by vorinostat. Changes in histone (H) acetylation were quantified using flow cytometry which is shown (for representative participant) as (A) fold change in mean fluorescence intensity (MFI) of antibody to acetylated (Ac) H3, Ac lysine (K) and Ac H4 in lymphocytes by size prior to, during and following vorinostat and (B) histograms of the change in MFI with antibody to Ac H3 following vorinostat. (C) PBMC were analysed by western blot using an antibody to Ac H3 and actin (as a control for total protein). A positive control (pos) of splenocytes from a mouse with acute myeloid leukemia treated with the HDACi panobinostat is also shown. (D) Fold change in acetylated (A) Histone 3 (red), (B) Lysine (orange) and (C) Histone 4 (blue) in total lymphocytes is shown for each study participant (solid circle) and the median (IQR) fold change above baseline is shown. *p<0.01, **p<0.001. Grey shaded box represents the time on vorinostat.

Changes in histone acetylation were measured by flow cytometry (for acetylated (Ac) H3, Ac lysine and AcH4) or western blot (Ac H3) (Figure 1A–C) and using flow cytometry we observed a statistically significant increase in Histone 3 and total lysine (K) acetylation during administration of vorinostat which returned to baseline following cessation of drug (**Figure 1D**). The time to peak acetylation for each patient was variable with 9 of 20 participants only achieving peak acetylation of H3 or lysine, after 7 days of drug.

**Figure 2:**
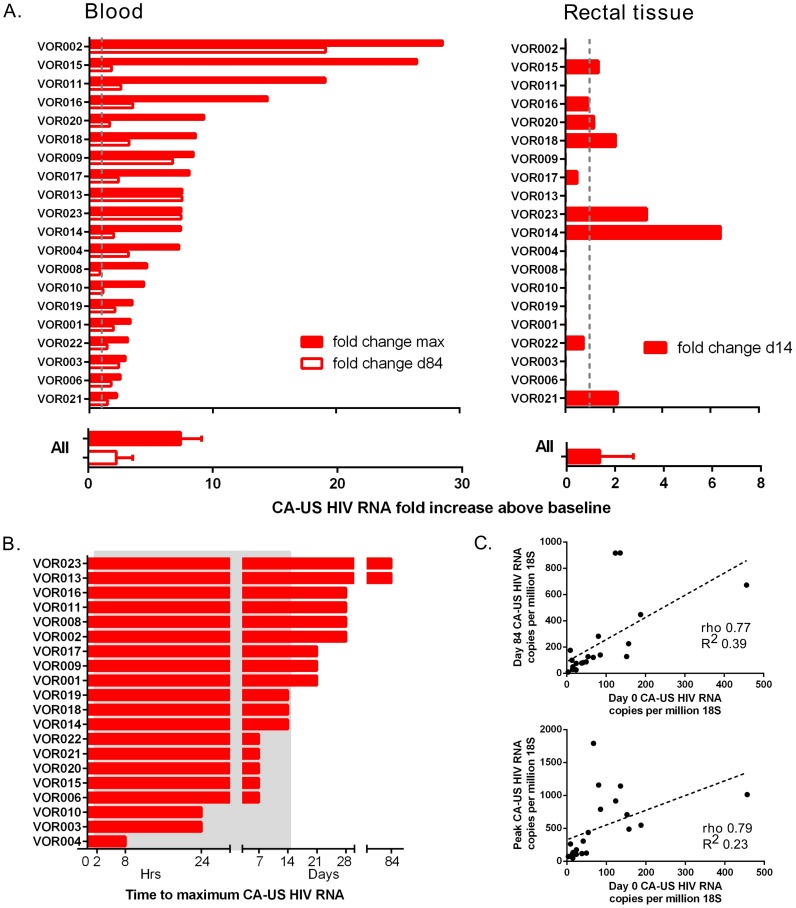
Individual changes in CA-US HIV RNA in blood and tissue. A) Fold change in CA-US HIV RNA following vorinostat in CD4+ T-cells from blood (left panel) and rectal tissue (right panel) compared to baseline. The maximum fold change in CA-US HIV RNA on study (solid column) and change at day 84 (open column) is shown for CD4+ T-cells from blood; and change at day 14 for rectal tissue is shown for each participant (upper panel) and the median (IQR) change for all participants (lower panel). The grey dashed line indicates 1-fold change. B) Time to reach maximum fold increase in CA-US HIV RNA for each participant. Grey shaded box represents the time on vorinostat. (C) Correlation between baseline CA-US HIV RNA and peak CA-US HIV RNA (left panel) and day 84 CA-US HIV RNA (right panel).

**Figure 3:**
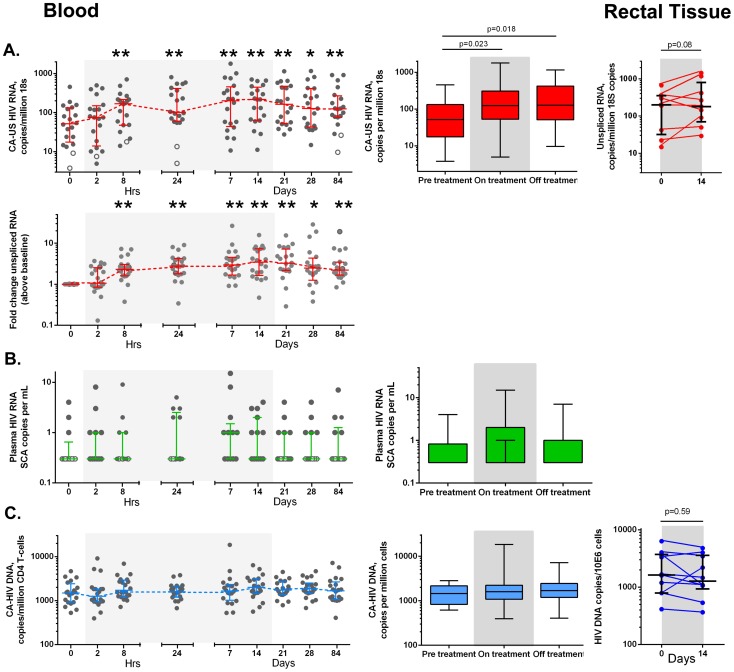
Effects of vorinostat on CA-US HIV RNA, HIV DNA and HIV RNA in blood and tissue. Changes in (A) CA-US HIV RNA (red), (B) plasma HIV RNA (green) and (C) HIV DNA (blue) is shown for each study participant (solid circle) and the median (IQR) at each time point in CD4+ T-cells from blood (left panel) and rectal tissue (right panel). Open circles represent data when at least one of the replicates were below the lower limit of detection (LLOD). The mean fold change in each parameter is also shown using a generalised estimating equation analysis (middle panel; boxes represent the median, 50^th^ and 75^th^ percentiles and whiskers represent the range). Grey shaded box represents the time on vorinostat. *p<0.01, **p<0.001.

There was a significant increase in CA-US HIV RNA in CD4+ T-cells from blood between baseline and the primary endpoint at day 14 (p<0.001). Intra-individual change in CA-US HIV RNA from baseline was significant in 90% (18/20) of participants on at least one time point during vorinostat dosing. The median fold change in CA-US HIV RNA from baseline to peak in CD4+ T-cells from blood was 7.4 (IQR 3.4, 9.1) and in rectal tissue from baseline to day 14 was 1.4 (IQR 0.8, 2.8; **Figure 2A**; individual fold changes shown in **Figure S2**). The time to peak change in CA-US HIV RNA in blood varied from 8 hours to 84 days (**Figure 2B**). CA-US HIV RNA at peak and day 84 correlated with baseline values (p<0.0001 for both; ρ = 0.23 and 0.39 respectively; **Figure 2C**). The increase in CA-US RNA was statistically significant by 8 hours after first dose and remained elevated throughout follow-up, including throughout the 70 day period after vorinostat dosing (p<0.001 for all time points for both comparison of raw data in copies per million 18s or when measured by fold-change, except day 28; **Figure 3A**). Using a generalised estimating equation (GEE) analysis, the mean fold change in CA-US HIV RNA relative to baseline at time points during vorinostat was 2.65 (95% CI 1.76, 3.52, p = 0.023) and at time points after vorinostat (study days 21, 28 and 84) was 3.00 (95% CI 2.16, 3.84, p = 0.018). There was a trend toward an increase between baseline and day 14 in CA-US HIV RNA in CD3+ T-cells from rectal tissue (p = 0.08; **Figure 3A**). There was no statistically significant correlation between changes in CA-US HIV RNA and changes in acetylation of H3, lysine and H4 (p>0.05 for all comparisons).

### Vorinostat did not induce a change in HIV RNA in plasma or in the frequency of latently infected cells

The detection of an increase in HIV RNA in plasma following vorinostat was measured in real time using a commercial assay (Roche) with a lower limit of detection (LLOD) of 20 copies/ml and on batched frozen plasma using a more sensitive assay that that had a LLOD of 0.3 copies/ml (single copy assay, SCA) [20]. Despite the significant increase in CA-US HIV RNA, we found no significant increase in plasma HIV RNA using the SCA (**Figure 3B**) or the commercial HIV RNA assay.

**Figure 4:**
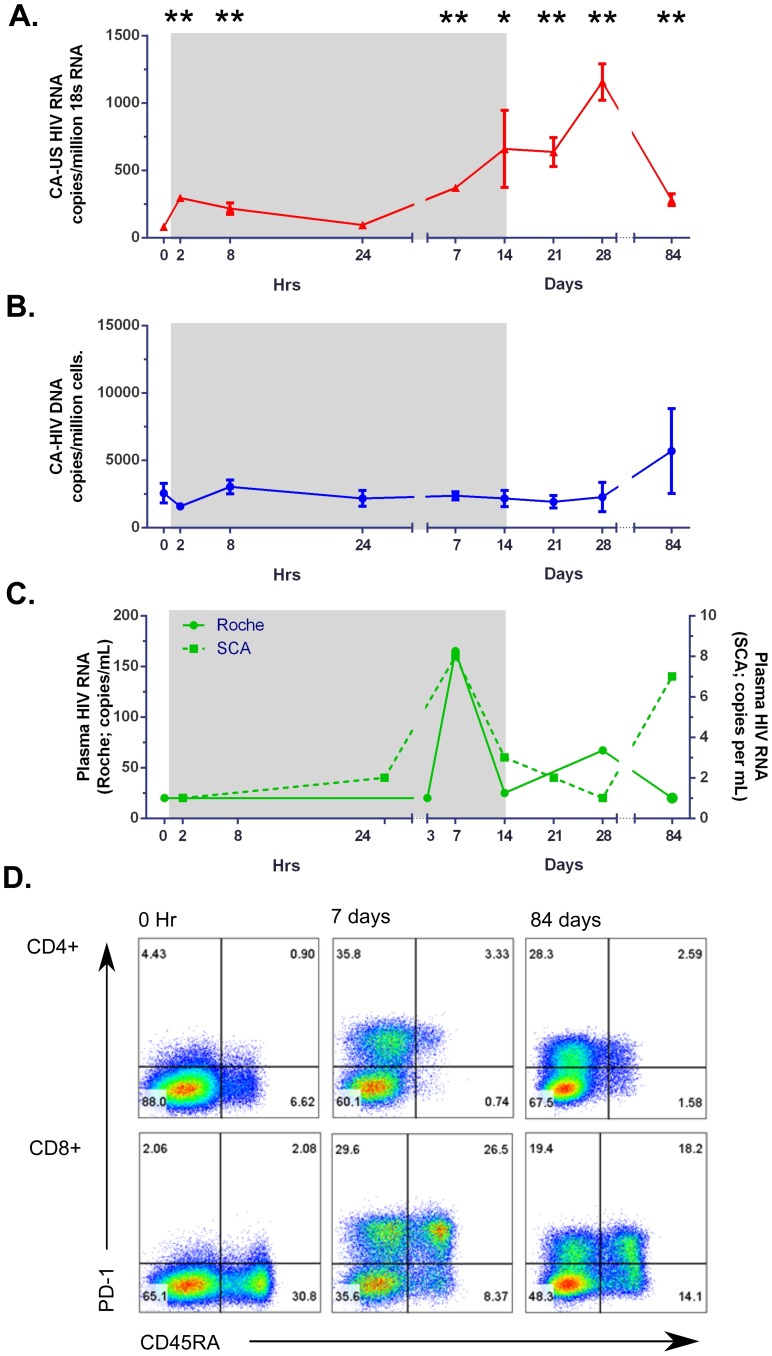
Changes in virological and immunological parameters from one patient with viral rebound on study. Changes in (A) CA-US HIV RNA (red), (B) plasma HIV RNA (green) and (C) CA-HIV DNA (blue) are shown as the mean± SD for replicates of CA-US HIV RNA and HIV DNA. Programmed death-1 (PD1) expression on CD4+ and CD8+ CD45RA- T-cells is shown. Grey shaded box represents the time on vorinostat. (D) Dot plot analysis of flow cytometry for co-expression of PD-1 and CD45RA on CD4+ (top panel) and CD8+ (lower panel) T-cells at baseline, after 7 days of vorinostat and at day 84 of follow up.

One participant had a qualitative increase (from <LLOD to >LLOD) in plasma HIV RNA at more than one time point during the study (peak 160 copies/ml at day 7) with a significant increase in CA-US HIV RNA (peak at day 28) and marked increase in PD-1 expression on CD8+ T-cells (**Figure 4**). This patient had had evidence of long term durable control of HIV RNA on ART with plasma HIV RNA <50 copies/ml, measured on 13 occasions over 5 years prior to enrolment in this study.

Consistent with the absence of production of HIV RNA in plasma, we found no change in HIV DNA (**Figure 3C**), no change in integrated DNA (n = 11; **Figure S1A**) nor inducible virus using a novel limiting dilution quantitative assay that detects multiply spliced (MS) tat and rev HIV RNA following activation with phorbol myristate acetate (PMA) and ionomycin (n = 6; **Figure S1B**). Finally, there was no change in HIV DNA in rectal tissue prior to and following vorinostat (**Figure 3C**)

### Vorinostat did not induce any significant increase in HIV-specific T-cells or immune activation but led to an increase in regulatory T-cells in blood

**Figure 5:**
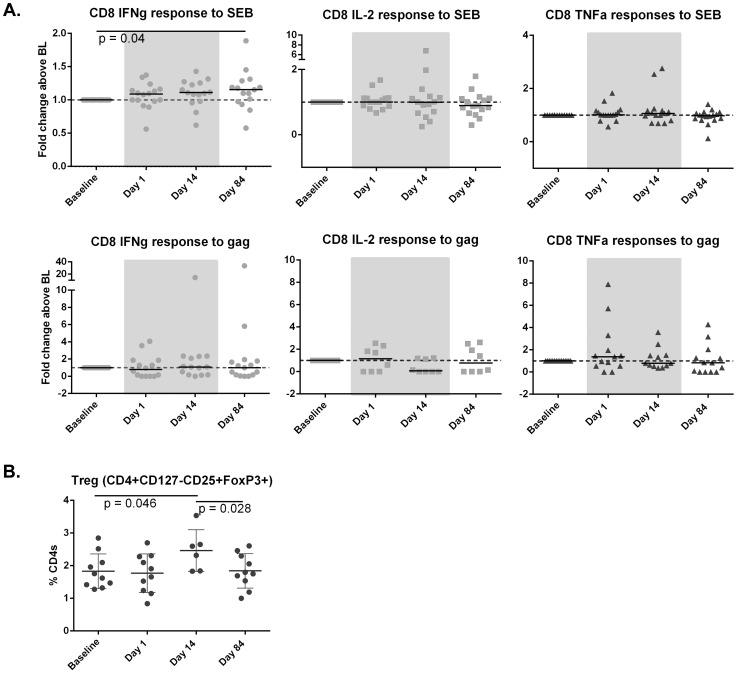
Vorinostat induced changes in the adaptive immune response. (A) SEB-specific (upper panel) and gag-specific (lower panel) CD8+ T-cells were quantified by intracellular cytokine staining and changes in (B) frequency of regulatory T-cells using flow cytometry. Significant changes between time points were determined using a Wilcoxon sign-rank test. P values<0.05 were considered significant. All statistically significant differences are shown.

Given there are no sensitive markers to determine HIV protein expression in vivo, we measured gag-specific T-cells as a strategy to detect any potential change in protein expression and/or the adaptive immune response following vorinostat. Gag-specific CD4+ and CD8+ T-cells did not increase following vorinostat, despite an increase in staphylococcal enterotoxin B (SEB)-specific interferon gamma (IFN-γ) producing CD8+ T-cells over the study duration measured by either fold change compared to baseline (p = 0.04; n = 11; **Figure 5A**) or the absolute percentage of CD8+ T-cells expressing IFN-γ (p = 0.04;). There was an increase in regulatory T-cells observed that returned to baseline following cessation of drug (**Figure 5B**). There were no changes in markers of immune activation and differentiation in blood and rectal tissue (**Figure S3**).

### Vorinostat induced dynamic changes in host gene expression in the 24 hours following the first dose of vorinostat

**Figure 6:**
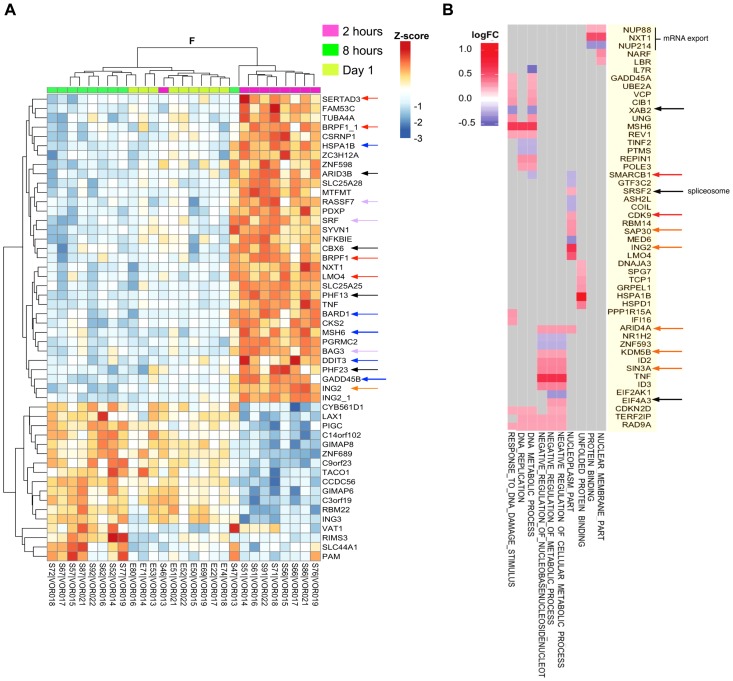
Vorinostat induced a transcriptional burst and chromatin perturbations that are recurrent with subsequent dosing. (A) ANOVA (F-test) heatmap of top 50 differentially expressed genes (DEGs) from matched donor supervised analysis (n = 9) comparing gene expression two hours (2 h), eight hours (8 h) and one day following the initial dose of vorinostat. Gene expression was adjusted for baseline expression and represented as a gene-wise standardized expression (Z-score), with p-values<0.05. DEGs are annotated with colored arrowheads: red, transcription coactivators and adaptors; blue, DNA damage and ER stress response; purple, apoptosis resistance; black, chromatin remodeling factors; orange, mSIN3a histone-deacetylase complex subunits. (B) Checkerboard map of DEG two hours after the initial dose of vorinostat compared to baseline showing the top 10 enriched pathways on the x-axis and leading edge analysis (gene members contributing most to enrichment) plotted along the y-axis. Scale represents log_2_ fold change where red corresponds to up- and blue down-regulated genes at two hours versus baseline. Genes associated with viral transcriptional activity are annotated with colored arrowheads: red, BAF component SMARCB1 (SNF5) and CDK9; black, splicesome and nuclear export proteins; orange, mSIN3A HDAC subunits.

We next used Illumina microarrays to characterize the kinetics and nature of host gene expression changes in whole blood following vorinostat administration and to determine whether there was a distinct transcriptional profile associated with changes in CA-US RNA. We observed highly significant changes in expression of multiple host genes compared to baseline at all time points including at two hours post vorinostat (**Figure S4**). HDACi compounds mediate dynamic changes in chromatin states by binding to HDACs, promoting open chromatin structure, DNA hypomethylation, and histone and non-histone protein acetylation which can lead to enhanced accessibility for the basal transcription machinery. This leads to a reduction in replication fork velocity and an increase in DNA replicative stress culminating in DNA damage and double strand DNA breaks [21], [22]. DNA double-strand break repair is essential for maintenance of genome stability following vorinostat administration. We were able to track temporal changes in gene expression associated with enhanced transcriptional activity and the DNA damage response (DDR) over the course of 24 h after the 1^st^ dose of vorinostat (**Figures 6A and 6B**) and subsequent time-points up to 70 days after the last dose.

At two hours following the first dose of vorinostat, we saw a burst in transcriptional activation including upregulation of sequence-specific DNA binding transcription factors YY1, Serum Response Factor (SRF) and SERTA Domain-Containing Protein 3 (SERTAD3), a potent co-activator of E2F responsive promoters [23] (**Figure 6A**). We also found upregulated expression of several Plant Homeo Domain (PHD) zinc finger proteins including Bromodomain and PHD Finger-containing protein 1 BRPF1, PHF13, PHF23, and Inhibitor of Growth family member 2 (ING2) [24], [25]. These proteins function as histone readers recognizingH3K4 methylation and recruit histone acetyl-transferase complexes involved p53/TP53cell cycle arrest (GADD45ACDKN2D) and DNA repair (RAD9A, MSH6, BRCA1 associated RING domain 1 BARD1, REPIN1, and POLE3) (**Figure 6B**) [22].

Heat shock proteins (HSP), HSP70B, HSP60, HSP40A3, and HSP90B1 associated with the unfolded protein response were also significantly up-regulated at this time point (**Figure 6B**), in parallel with HDAC1 and HDAC2, the multi-subunit HDAC-mSin3a complex (SIN3A, SAP30, ING2, ARID4A, ARID5A, KDM5B) and several chromodomain proteins, which are part of the Polycomb Repressive Complex 1 (PRC1) (orange arrowheads, **Figure 6B**). Importantly, components of the splicesome and nuclear export proteins were upregulated at two hours indicating functional splicing machinery (black arrowheads **Figure 6B**).

Since previous studies found that a switch in the subunit composition of the switch/sucrose-non fermenting (SWI/SNF) ATP-dependent chromatin remodeling complexes between BRG1-Associated Factor (BAF) and PBAF was required for nucleosomal repositioning and initiation of transcription at the HIV LTR, we searched for differential expression of variant subunits exclusive to PBAF complexes [26]–[28]. SWI/SNF complexes control the nucleosome landscape at active promoters and are required for HIV Tat transactivation [29]. Interestingly, we found that two core components of the SWI/SNF ATP-dependent BAF chromatin remodeling complex, SWI/SNF related, matrix associated, Actin dependent Regulator of Chromatin, subfamily D1 (SMARCD1), member 1 BAF60, and SMARCB1 (SNF5/INI1) were down-regulated two hours following vorinostat (red arrowhead **Figure 6B**). SNF 5/INI1 is an essential HIV host cell factor involved in the integration of viral cDNA into active genes [30]. Its expression interferes with viral replication and knockdown has been shown to increase expression of HIV 2LTR circles, a dead end product of HIV replication [30]. In addition, recent studies suggest that loss of SNF5 activity results in the disruption of nucleosome occupancy at transcriptional start sites (TSS) of gene promoters resulting in the upregulation of E2F target genes associated with cell cycle and proliferation [28].

Vorinostat induced transient expression of both the serine/threonine kinase CDK9 (red arrowhead **Figure 6B**) and CCNT2, core subunits of the positive transcription elongation factor b (P-TEFb), and AFF4 and ELL, components of the super elongation complex (SEC) (**Table S4**) [31], [32].

We also found vorinostat induced expression of BRD2, a member of the bromodomain and extraterminal (BET) protein family that can compete with TAT for binding to P-TEFb and suppress HIV transcription in latently infected cells (**Table S4**). We did not see differential expression of CycT1 that specifically interacts with Tat as part of the SEC.

The rapid up-regulation of HDACs and chromatin co-repressor complexes two hours after the initial dose of vorinostat result in restoration of the normally repressive chromatin architecture leading to a shutdown of the transcription of early response genes by 8 hours that was maintained at 24 hours after vorinostat administration (**Figure 6A**) [21]. Most importantly, TNF-α and NF- κB which can promote HIV replication [10] were strongly upregulated at two hours after vorinostat and turned off by 8 hours. This is supported by the downregulation of CCAAT/enhancer binding protein (C/EBP), an important transcriptional amplifier of inflammatory response genes [33], and consistent with the anti-inflammatory effects of HDACis.

### Changes in host gene expression and induction of CA-US HIV RNA

**Figure 7:**
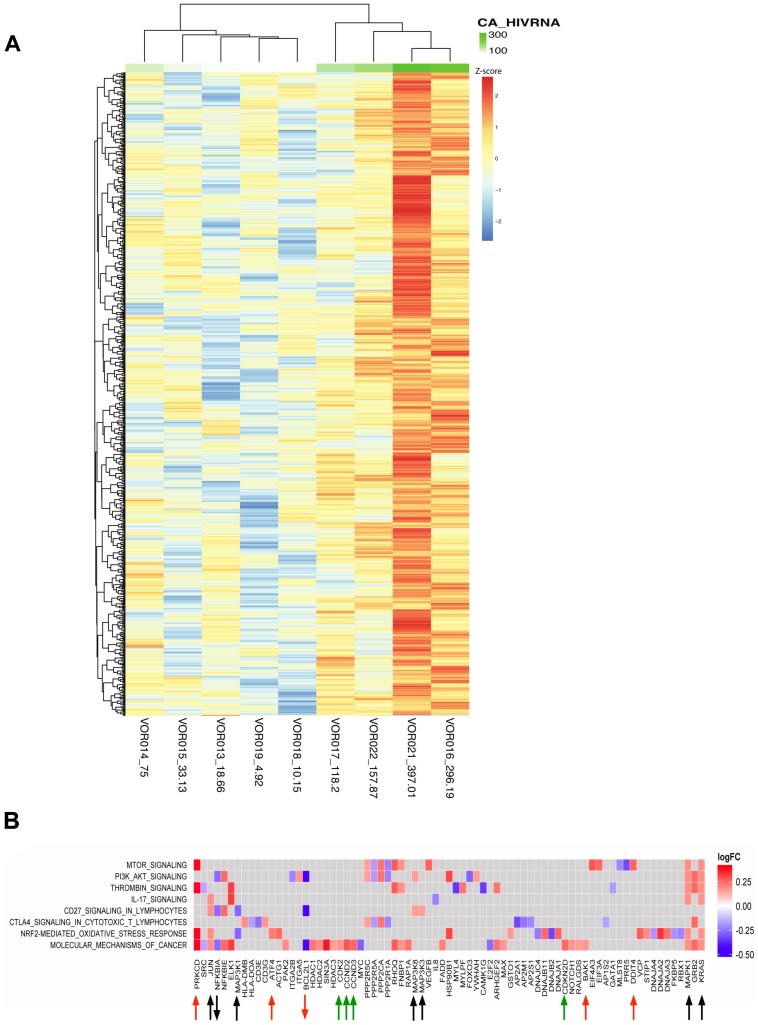
Changes in host genes were associated with an increase in CA-US HIV RNA after vorinostat. (A) Heatmap showing the fold change in gene expression using linear regression analysis between CA-US HIV RNA and DEG at two hours following the initial dose of vorinostat (n = 9). CA-US HIV RNA is plotted as a continuous variable (ranging from low to high – light green to dark green) and correlated with distinct gene expression profiles. The copy number of CA-US HIV RNA per million cells two hours following vorinostat for each participant is listed next to the patient identification code at the bottom of each column. The top 50 regression features (of a total of ∼2000 at nominal p-value<0.05) are shown. (B) Pathway analysis was performed on the regression features and a checkerboard map showing the top common enriched pathways on the y-axis and leading edge analysis (gene members contributing most to enrichment) plotted along the x-axis. Up- and down-regulated genes at two hours versus baseline are plotted as log_2_ fold change (FC); red squares correspond to upregulated gene expression and blue downregulated gene expression. Genes associated with MAPK signal transduction pathways are annotated with black arrows and cell cycle regulators annotated with green arrows. Genes associated with the ER stress response and apoptosis are annotated with red arrows.

Using linear regression, we observed a strong correlation between changes in CA-US HIV RNA with the transcriptional profile and pathways observed at two hours with 2201 genes differentially expressed with a p<0.05 (**Figure 7A**). Pathway enrichment analysis showed upregulation of MAPKs that activate both the stress-activated protein kinase (SAPK), JNK kinase pathways and extracellular signal-regulated protein kinase (ERK2) and NF-κB pathways, (black arrows **Figure 7B**). These pathways play a key role in upregulating AP-1 activity and T cell activation and differentiation and correlated with an increase in CA-US HIV RNA (**Figure 7B**). The expression of cyclin dependent-kinase subunit 2 (CKS2), CDK2, and CCND2 and CCND3 regulating G1-S phase transition at two hours post vorinostat was counterbalanced by the upregulation of inhibitors of cyclins CDK2 and CDK4 holoenzymes, CDKN1A,and CDKN2D p19^ink4D^ respectively, and GADD45A associated with p53-dependent cell cycle G1 phase arrest (green arrows) (**Figures 7B**). In addition, gene expression associated with the ER stress response and p53-mediated apoptosis also correlated with the increase in CA-US HIV RNA, (red arrows).

### Similar host gene changes were observed two hours after the first and seventh doses of vorinostat

**Figure 8:**
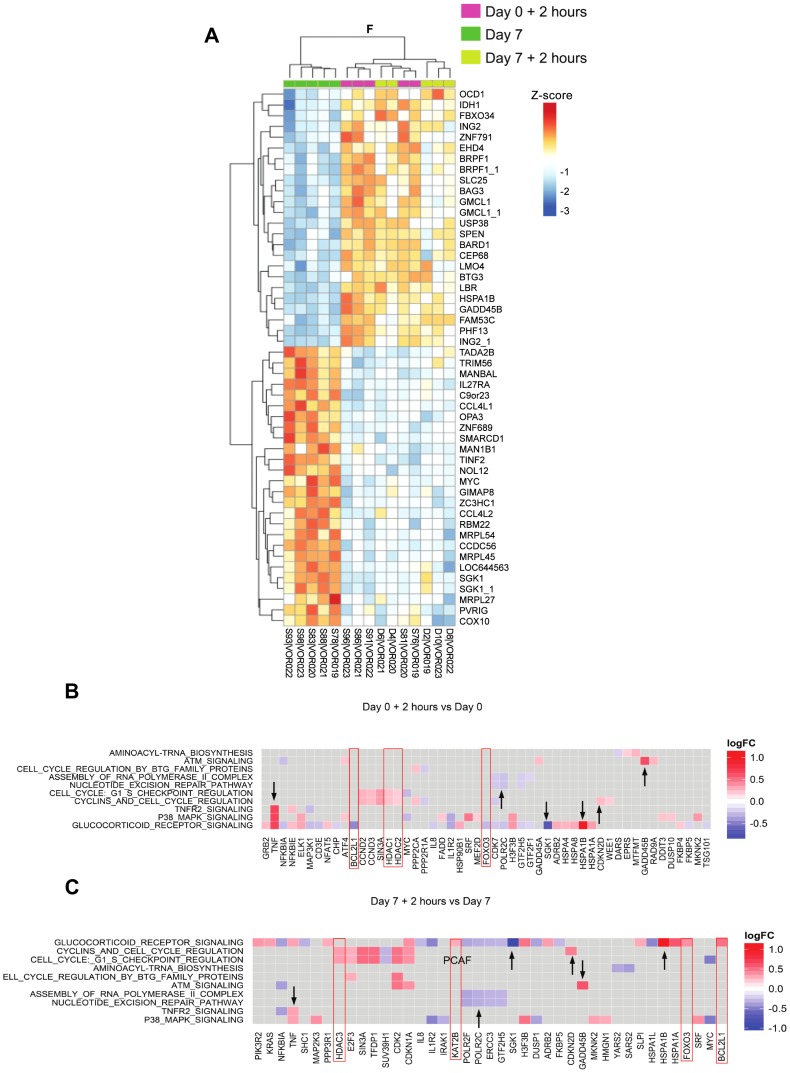
Similar gene expression and pathway activity after the 1st and 7th daily dose of vorinostat. (A) ANOVA (F-test) heatmap of top 50 DEGs from matched donor supervised analysis (n = 5) comparing gene expression at 2 hours following the first dose (day 0+2 hours), day 7 (7d; prior to the seventh dose), and two hours following the seventh dose (day 7+2 hours). Gene Expression was adjusted for baseline expression and represented as a gene-wise standardized expression (Z-score), with p-value<0.05. The heatmap shows hierarchical clustering of day 0+2 hour and day 7+2 hour timepoints for each subject indicative of highly congruent gene expression profiles. Checkerboard maps of DEG (B) two hours following the initial dose of vorinostat compared to baseline and (C) two hours following the seventh dose of vorinostat compared to prior to the seventh dose. The top 10 common enriched pathways are shown on the y-axis and leading edge analysis (gene members contributing most to enrichment) is plotted along the x-axis. Scale represents log_2_ fold change. Genes up- or down-regulated similarly between the two time-points are indicated by black arrowheads whereas genes highlighted in red boxes are inversely or differentially regulated between time points.

Given it remains unclear whether repeated doses of vorinostat induce similar upregulation of host gene, or HIV RNA expression [19], we compared changes in the gene expression two hours following the first and two hours following the seventh dose of vorinostat. Similar responses in gene expression and pathway activity were seen in four of the five subjects (**Figure 8** **and Figure S5**). There were some differences in genes associated with cell cycle arrest and survival (e.g. CDKN1A (p21), BCL2L and FOXO3) suggesting a shift from the pro-apoptotic DDR seen after the first dose to an oxidative stress response and cell survival after the seventh dose (**Figures 8B and 8C**).

We also compared changes after the first and seventh doses using gene set enrichment specific to different peripheral blood mononuclear cells (PBMC) subsets. We applied a method for reconstruction of subset-dependent gene expression modules using gene set enrichment and subset-specific network analysis. These cell subset-dependent modules are referred to as Nakaya modules, where cell subset specific gene activity is visualized in the radial plot shown in **Figure S5C**. The radial plot shows a wedge of color that points outward (increased expression) or inward (decreased expression). We found similar enrichment in gene expression for both the first and seventh doses in T cells, B cells, monocyte and plasmacytoid dendritic cells (pDCs) and significant downregulation of natural killer (NK) cell activity. Notably, the myeloid dendritic cell (mDC) population was significantly enriched in downregulated genes after the seventh dose only.

### Vorinostat was associated with persistent changes in host gene expression

**Figure 9:**
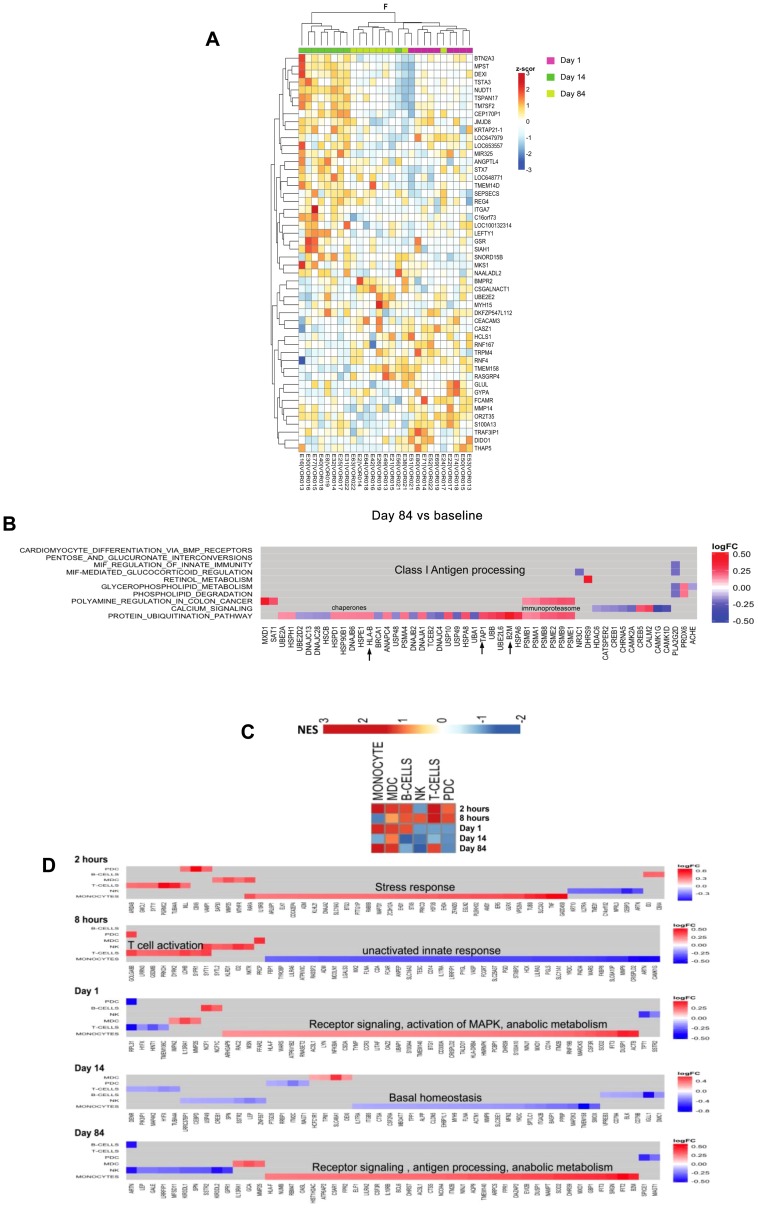
Changes in gene expression over the duration of study with most early changes occurring in T-cells. (A) ANOVA (F-test) heatmap of top 50 differentially expressed genes (DEGs) from matched donor supervised analysis (n = 9) comparing gene expression one, 14 and 84 days following the initial dose of vorinostat. Gene Expression was adjusted for baseline expression and represented as a gene-wise standardized expression (Z-score), with p-values<0.05. (B) Checkerboard map of differentially expressed genes 84 days after the initial dose of vorinostat (70 days post cessation of drug) compared to baseline showing the top 10 enriched pathways using gene subset enrichment analysis (GSEA) on the y-axis and leading edge analysis (gene members contributing most to enrichment) plotted along the x-axis. Scale represents log_2_ fold change where red corresponds to up- and blue down-regulated genes respectively. (C) Pathway heatmap illustrates enrichment of gene expression in PBMC subsets at different timepoints compared to baseline. Red and blue represent up and down regulated expression of gene subsets respectively. (D) Checkerboard map of DEG at each timepoint compared to baseline. The cell subsets (modules) are plotted on the y-axis and gene members contributing to enrichment plotted on the x-axis. Scale represents log_2_ fold change. Red and blue boxes represent up and down gene regulation respectively. mDC = myeloid cells; pDC = plasmacytoid dendritic cells; NK = natural killer cells.

Given that we observed persistent changes in CA-US HIV RNA out to 84 days (**Figure 2**), we asked whether significant changes in host gene expression also persisted and indeed found distinct changes in host gene expression through day 84 (**Figure 9A** **, S4**). Comparing differences in gene expression between day 1, day 14 and day 84 normalized to baseline (F test; **Figure 9A**), we saw a subset of differentially expressed genes after 14 days of vorinostat administration that reflected cellular detoxification processes whereas long term changes seen at day 84 (70 days after discontinuation of vorinostat) were significantly associated with protein ubiquitination and upregulation of MHC Class I antigen presentation (**Figure 9B**). Genes upregulated early (2 hours to one day) after vorinostat administration occurred mostly in T cells while at day 84, monocytes and mDCs showed the bulk of upregulated genes (**Figures 9C and 9D**).

## Discussion

In this study of 14 days of continuous daily dosing, vorinostat was safe, relatively well tolerated and induced a sustained increase in CA-US HIV RNA in CD4+ T-cells from blood in the majority of participants, suggesting that the latent HIV provirus in most virally suppressed adults is susceptible to HDAC inhibition.

Despite the significant increase in CA-US HIV RNA following vorinostat, there was no significant change in plasma HIV RNA in all but one subject. This may be because vorinostat induced an increase in HIV transcription, as measured by CA-US HIV RNA, but subsequent blocks in virus production were not reversed, including synthesis or export of multiply spliced HIV RNA from the nucleus to the cytoplasm [34] and/or inhibition of translation by cellular microRNA [35]. Alternatively, this may be due to induction of HIV transcription in only a small subset of latently infected cells [9], [36] or the production of incomplete short or read through transcripts, as described in ex vivo models [12], [37]. Recent studies using resting CD4+ T-cells isolated from HIV-infected patients on ART stimulated with vorinostat and other HDACi, have also demonstrated that production of free virus from latently infected cells was rarely observed, even when an increase in CA-US HIV RNA was clearly detected [9], [11], [12]. Our in vivo findings are consistent with these in vitro observations although it is important to note that we did see evidence for the upregulation of genes associated with splicesome assembly and nuclear export of mRNA at 2 h suggesting functional splicing machinery (**Figure 6B**).

HDACi have a broad range of effects on both the adaptive and innate immune responses, and have been shown to induce both the number and function of regulatory T-cells [38], as we show in this study. Vorinostat has also been shown to reduce anti-CD3 mediated T-cell proliferative responses in a dose dependent fashion [39], although interestingly, in this study we observed a significant increase in mitogen-specific IFN-γ CD8+ T-cell responses over the duration of follow-up. However, despite these diverse changes in global T-cell function, we still saw no change in gag-specific T-cells in this study most likely because translation and HIV protein expression were insufficient to induce priming or recall of HIV-specific T-cells.

The timing and magnitude of increase in both CA-US HIV RNA and histone acetylation were highly variable, consistent with previous reports of HDACi in patients with malignancy [40]. The lack of a statistically significant relationship between changes in HIV CA-US RNA and any of the markers of acetylation supports recent data suggesting that the activity of HDACi in stimulating HIV transcription may not occur by the direct effects on histone acetylation, but via effects on other proteins such as the release of free P-TEFb from the inhibitory complex 7SK snRNP [31]. HDACi have a wide ranging effect on acetylation of both histone and non-histone proteins [41] and therefore other, yet unidentified, proteins may also have played a role in activating HIV transcription.

Using gene array we showed a strikingly consistent change in expression in multiple genes in all subjects as quickly as two hours following the initial dose. Although it is difficult to ascribe direct effects of vorinostat on specific pathways associated with chromatin remodeling, transcriptional activation, spliceosome activity or mRNA export with expression of CA-US HIV RNA, our analysis of host gene expression showed that vorinostat can effectively promote or prime a favorable epigenetic environment for HIV transcription. The coordinated and highly consistent changes in host gene expression that favour HIV transcription, also argues against random, non-specific read-through [12]. However, we acknowledge that distinguishing between read-through transcripts and viral CA US-RNA would need to be measured directly on these samples to confidently exclude the possibility of only read-through transcription occurring. Hence, the mechanisms by which vorinostat and perhaps other HDAC inhibitors affect HIV reactivation might be multi-factorial, with the drug directly stimulating HIV transcription and indirectly altering expression of other host genes that affect this process. Induction of HIV transcription is a critical first step in activating virus production from latency. Therefore, HDACi such as vorinostat are likely to play an important role in combination activation strategies given the significant synergism demonstrated when combining HDACi with other agents, such as protein kinase C activators and/or BET inhibitors, leading to the induction of virus production from latently infected cells from HIV-infected patients on ART ex vivo [12], [42], [43].

It remains unclear what the optimal dosing of vorinostat should be in activating HIV transcription. A recent study using an unconventional dosing schedule of 3 days per week of vorinostat demonstrated an increase in CA-US HIV RNA in 3 of 5 participants following multiple but intermittent doses of drug [19]. The authors noted a reduced response in production of CA-US HIV RNA after the 11th and 22nd doses, compared to the first dose, although there were too few patients and time points studied to determine whether there were any statistically significant differences in the magnitude of the changes observed [19]. In our study, multiple doses of vorinostat were clearly well tolerated and there were similar robust changes in host gene expression following the first and seventh doses. Although an increase in CA-US HIV RNA was observed in all participants who responded within 8 hours, in some participants the maximum fold increase in CA-US HIV RNA was only observed after 7 or 14 doses, arguing that multiple doses of vorinostat may be needed to maximize any changes in HIV transcription. Whether this is the case for other HDACi remains unknown. There are now more potent HDACi than vorinostat in clinical trials for malignancy and activation of latent HIV [44], that have significantly enhanced potency in activating latent HIV infection in vitro, including panobinostat, entinostat and romidepsin [11], [12], [44], [45], and these more potent agents will hopefully show greater potency than vorinostat in vivo [44].

An unexpected finding in our study was the unique changes in host gene expression detected out to day 84–70 days off vorinostat when compared to baseline. This was despite a return to baseline levels of histone acetylation upon cessation of drug following the sustained increase whilst on vorinostat. It will be important to determine if the expression of IFN inducible genes (ISGs) associated with antigen processing and Class I antigen presentation at day 84 reflects long-term epigenetic changes at the viral LTR that might facilitate virus reactivation and/or that promote innate immune responses. These changes may explain the observed increased CA-US HIV RNA expression to day 84 in this study. The correlation between baseline and both peak and day 84 levels of CA-US HIV RNA suggests activation of transcription may be more efficient in patients with higher levels of basal HIV transcription.

Latently infected resting memory CD4+ T-cells represent the most significant barrier to HIV eradication. In this study we demonstrated that although short-course vorinostat clearly induced CA-US HIV RNA, there was no evidence of production of free virus or elimination of latently infected cells. Vorinostat may be a feasible component of larger HIV eradication studies given its tolerability and the induction of HIV transcription in the majority of participants without the need for *ex vivo* screening, although the prolonged changes in host gene expression require careful long term follow up. The lack of change in the number of infected CD4+ T-cells suggests that vorinostat did not impact the size of the HIV reservoir despite a clear effect on HIV transcription. Additional interventions will be needed to efficiently induce virus production and ultimately to eliminate latently infected cells.

## Materials and Methods

### Participants and study procedures

We recruited twenty adults aged 18–60 years receiving at least three antiretroviral agents, with a plasma HIV RNA <50 copies per mL for at least three years (excluding single viral ‘blips’), a CD4+ T-cell count >500 cells/µL and documented subtype B HIV-1 infection. We excluded individuals with significant acute illness, hepatic or cardiac disease, diabetes, malignancy, transplantation or recent use of immunomodulatory agents. We initially excluded patients receiving protease inhibitor regimens but this was modified once further data had become available and subsequently there were no exclusions on the basis of antiretroviral regimen. Participants provided informed consent and The Alfred Human Research Ethics Committee approved the study. The study is registered at ClinicalTrials.gov (NCT01365065).

Participants received vorinostat 400 mg orally once daily for 14 days. Blood was collected at 0, 2, 8 and 24 hours, and on days 7, 14, 21, 28 and 84. Rectal biopsies were performed at baseline and on day 14. Participants were monitored for clinical and laboratory adverse events, graded according to the National Cancer Institute Common Terminology Criteria for Adverse Events (Version 4.0). A Data Safety Monitoring Board reviewed safety and tolerability data after the first and tenth participants had completed dosing.

### Study endpoints

The primary study objective was to evaluate the effect of vorinostat on HIV transcription. We measured CA-US HIV RNA because this is the first product of HIV transcription and is required to ultimately synthesise MS-HIV RNA, viral proteins and single stranded viral RNA needed for new virion production [46], [47]. Secondary efficacy endpoints were a sensitive measure of plasma HIV RNA with a lower limit of detection (LLOD) of 0.3 copy per mL [20] cell associated and integrated HIV DNA (a measure of the total number of infected CD4+ T-cells); and histone (H3, H4 and lysine) acetylation (a pharmacodynamic marker of vorinostat activity). Safety endpoints were plasma HIV RNA measured using a commercial assay with a LLOD of 20 copies per ml (TAQMAN v2, Roche), adverse events, serious adverse events, dose limiting toxicity, CD4+ T-cell count and plasma trough concentrations of antiretroviral agents. Because HDACi can activate DNA viruses we quantified cytomegalovirus (CMV) and Epstein-Barr virus (EBV) DNA at baseline and day 28.

### Measurement of trough concentrations of antiretroviral agents

Trough concentrations of non-nucleoside reverse transcriptase inhibitors (NNRTI) or protease inhibitors (PI) in blood were performed at baseline and day 14 using a validated high performance liquid chromatography (HPLC) assay.

### Histone acetylation

Thawed PBMC were permeabilised, fixed with 90% methanol, then stained using antibodies to acetylated (Ac) histone (H)3, and Ac lysine (Millipore, Billerica, MA) and Ac H4 (kind gift from Dr Jeff Lifson, National Cancer Institute Frederick, Frederick, MD) and associated isotype controls with secondary staining with either goat-anti-rat PE or goat anti-mouse-FITC (Invitrogen). Lymphocytes were gated by size and data expressed as MFI above isotype control. Fold changes were determined by comparison of MFI at each time point above baseline MFI.

### CA-US HIV RNA and HIV DNA in CD4+ T-cells

CD4+ T-cells were isolated from stored peripheral blood mononuclear cells (PBMC) using a CD4+ T-cell isolation kit and magnetic-activated cell sorting (MACS) columns (Miltenyi Biotec, Teterow, Germany; purity >95%) and RNA and DNA extracted (Allprep isolation kit, Qiagen). For quantification of CA-US RNA, a semi-nested real time quantitative (q) PCR was used with a first round amplification of 15 cycles to ensure that following second round amplification the assay was in the linear range between 1 to 46,000 input copies, as previously described by Pasternak et al [48]. The second round used primers to gag [46]. HIV RNA copy numbers were standardised to cellular equivalents using an 18s RNA real time LUX PCR primer set (Invitrogen). The LLOD for CA-US HIV RNA was 1 copy per well. PCR amplification of cDNA for CA-US HIV RNA was performed in quadruplicate with an intra-assay coefficient of variation (CV) of 32%. In all assays, a no reverse transcriptase (RT) control was used. If there was any amplification from the no RT control, ie. evidence of DNA contamination, a second stored sample was re-extracted. If contaminating DNA persisted, the reading was excluded. Repeat extraction was required for only 2 of a total of 200 samples analysed for this study. HIV DNA was quantified as previously described [49]. PCR for HIV DNA was performed in triplicate for all samples with an intra-assay CV of 21%. Integrated DNA was measured in total CD4+ T-cells as previously described [50].

### HIV RNA and HIV DNA from rectal tissue

Single cell mononuclear cell suspensions were obtained from rectal biopsies [51]. Cells were stained using a cocktail of antibodies to CD3, CD8, CD45 and CD4 (Multitest, BD Biosciences, Franklin Lakes, NJ) and sorted for CD45+CD3+ cells using high speed flow cytometry (FACSAria, BD Biosciences). CA-US HIV RNA and HIV DNA were quantified as above.

### Inducible virus quantification by Tat Rev Inducible Limiting Dilution Assay (TILDA)

CD4+ T cells were isolated from PBMCs from study participants by negative magnetic selection (StemCell), and stimulated with phorbol myristate acetate (PMA; 100 ng/mL) and ionomycin (1 µg/mL) for 12 h. Serial dilutions of the stimulated cells were placed in a 96 well plate directly in RT-PCR buffer using 1 in 10 dilutions (4 times) and with 24 replicates at each dilution. MS HIV RNA was quantified by semi nested real time PCR with primers in tat and rev as previously described [52] with some minor modifications. The frequency of positive cells was calculated using the maximum likelihood method [53] and this number was then expressed as a frequency of cells with inducible MS HIV RNA per million CD4+ T-cells.

### Phenotyping for immune activation

Immune activation and differentiation were quantified as previously described [54]. In brief, one million thawed PBMC were stained with either an activation or differentiation panel for 15 minutes at 37°C prior to fixation in formaldehyde. Both panels included CD3 V450 (Becton Dickinson); CD4 PE-Texas Red (Invitrogen); CD8 Qdot605 (Invitrogen). Activation panel included HLA-DR FITC; PD-1 AF647; CD38 PE; CCR5 PE-Cy5; 45RA PE-Cy7 (all Becton Dickinson); CCR7 APC eFluor-780 (eBioscience). Differentiation panel included CD45RA PE; CD28 PE-Cy5; CCR7 PE-Cy7; CD31 FITC (all Becton Dickinson); CD57 AF647 (Biolegend); CD27 AF780 (eBioscience). For Tregs, PBMC were surface stained with CD4 PerCP, CD127 PE and CD25 FITC (all Becton Dickinson) followed by intracellular staining using eBioscience FoxP3 staining kit and FoxP3 APC as per manufacturer's instructions. Data was acquired on a BD LSR-Fortessa and analysed using FlowJo version 10.

### Intracellular cytokine staining

Thawed PBMC were rested for 12 hours prior to stimulation of 1.5 million cells each with Brefeldin A (Sigma Aldrich) and either gag peptides (1 ug/peptide/mL; NIH AIDS reagent program); Staphylococcal enterotoxin B (SEB; 1 ng/mL) or Dimethyl sufoxide (DMSO) for 6 hours. Cells were then surface stained with CD3 AlexaFluor700, CD8 Pacific Blue, CCR7 PE-CF594, PD-1 PE-Cy7 (all BD Biosciences), CD4 Qdot 605 (Invitrogen), CD45RA Brilliant Violet 650, CD19 Brilliant Violet 510 (Biolegend), CD27 APCe780, and aqua fluorescent reactive dye (Invitrogen), permeabilised with Saponin and stained intracellularly with IL-2 PerCP-Cy5.5, IFNγ APC and TNFα Alexa Fluor 488 (all BD Biosciences) prior to fixation. Cells were acquired within 24 hrs using a BD LSR-II and analysed using FlowJo version 9 and 10.

### Gene microarray and bioinformatic analyses

Blood was collected directly into Paxgene tubes and cells lysed for RNA extraction as per manufacturer's instructions (Qiagen, Valencia, CA). Reverse transcription reactions were performed to obtain cDNAs which were hybridized to the Illumina Human HT-12 version 4 Expression BeadChip according to the manufacturer's instructions and quantified using an Illumina iScan System. The data were collected with Illumina GenomeStudio software. Analysis of the genome array output data was conducted using the R statistical language [55] and the LIMMA statistical package [56] from Bioconductor [57]. First, arrays displaying unusually low median intensity, low variability, or low correlation relative to the bulk of the arrays were tagged as outliers and were discarded from the rest of the analysis. Quantile normalization followed by a log2 transformation using the Bioconductor package LIMMA was applied to process microarrays. The LIMMA package was used to fit a linear model to each probe and to perform a (moderated) Student's *t* test on various differences of interest. For data mining and functional analyses, genes that satisfied a p-value (0.05) were selected. Probes that did not map to annotated RefSeq genes and control probes were removed. When indicated, the expected proportion of false positives, the false discovery rate (FDR), was estimated from the unadjusted p-value using the Benjamini and Hochberg method [58].

The full dataset was composed of 9 patients with 8 time points each. Samples were stratified into the following groups: Day 0 (baseline), 2 hours, 8 hours, Day 1, Day 14 (all on vorinostat); and Day 84 (off vorinostat). One set of analyses compared each time point to baseline (to show the persistent effect of vorinostat over time). The other set compared baseline gene expression at 2 hours, 8 hours and 1 day (to isolate the early effects of vorinostat following the initial dose). In 5 individuals, the additional time points including Day 7 and Day 7+ 2 hours were collected to determine if changes seen 2 hours after the first dose were the same as those seen at 2 hours following the 7^th^ dose.

Heatmaps of genes differentially expressed between different groups and baseline were produced (**Figure S3**). Two ANOVA (F-test) heatmaps comparing the groups of interest: 2 hours, 8 hours, Day 1 (**Figure 6A**) and Day 0+2 hours, Day 7, Day 7+2 hours (**Figure 8A**) were produced. The top 50 statistically significant genes are shown as symbols and plotted on the row names of the heatmaps. Gene expression within each heatmap is represented as gene-wise standardized expression (Z-score), with p-value<0.05 chosen as the significant level.

Gene Set Enrichment Analysis (GSEA) [59] was performed on the various contrasts of interest. GSEA is a statistical method to determine whether members of a particular gene set preferentially occur toward the top or bottom of a ranked-ordered gene list where genes are ranked by the strength of their association with the outcome of interest. More specifically, GSEA calculates a net enrichment score (NES) that reflects the degree to which a set of genes is over-represented among genes that are differently expressed. We apply a nominal p-value cutoff of 0.05 when plotting the top enriched pathways on a checkerboard figure. The NES and p-value rankings usually go hand in hand i.e.: the top 10 NES pathway scores equate with the top 10 significant p-values). Since gene expression is tightly regulated, we do not apply statistical cutoffs on the actual FC's of genes when performing pathway enrichment. We try to include as many genes as possible to capture enriched and coregulated transcripts within a pathway that are an indication of relative pathway activity. The significance of an observed NES is obtained by permutation testing: resorting the gene list to determine how often an observed NES occurs by chance.

Leading Edge analysis was performed to examine the particular genes of a gene set contributing the most to the enrichment. Two different databases were used: Ingenuity Pathway Analysis software (Ingenuity H Systems, www.ingenuity.com) was used to mine canonical pathways while MSigDB (www.broadinstitute.org/msigdb; Broad Institute, Cambridge, MA) was used to mine chromatin and splicing pathways. A list of significant pathways ranked by p-value and NES is provided (**Dataset S1**).

Linear regression analysis was performed between CA-US HIV RNA at 2 hours and the gene expression 2 hours after the first dose of vorinostat versus baseline (n = 9). CA-US HIV RNA was plotted as a continuous variable and correlated with distinct gene expression profiles at low and high levels of CA-US HIV RNA (**Figure 7A**). About 2000 features passed the p-value cut off of <0.05. Pathway analysis was performed on the regression features and a checkerboard figure with some of the top resulting pathways was produced (**Figure 6B**).

A radial plot (**Figure S4C**) illustrating the different enrichment scores in PBMC cell specific subsets [60] between samples collected at day 0+2 hours and day 7+2 hours is shown. Checkerboard figures were used as a representation of the pathway analysis results representing the top genes and the top pathways for a specific contrast. Checkerboard plots show the top 10 enriched pathways on one axis and leading edge analysis (genes contributing to that enrichment) on the corresponding axis. This approach allows quick visualization of what genes are up regulated (red) or down regulated (blue) in the respective pathway at the specified contrast. Checkerboard analysis was also performed on a cell subset level [60] and the same plots generated together with a subset enrichment heat map displaying the contrasts on the x-axis and the subset on the y-axis (**Figure 9C**).

### Statistical methods

A sample size of 20 patients gave 80% power to detect an increase in CA-US HIV RNA of 40 copies/million CD4 T cells (primary endpoint) and an increase in plasma HIV RNA using the single copy assay of 0.4 log (secondary efficacy endpoint) at a p<0.05 level of significance. Categorical variables were summarised using frequency and percentage whilst continuous variables were summarised using mean and standard deviation (SD) or median and inter-quartile range (IQR) as appropriate. Spearman rank correlation coefficients were calculated between virologic and immunologic measurements. Intra-individual comparisons of CA-US RNA and HIV DNA between baseline and post-baseline time points were performed using parametric summary measures of replicate PCR data and a parametric paired t-test as PCR replicate data were derived using a standard curve and thus approximated a normal distribution. Bonferroni adjustment was made for multiple comparisons.

Whilst we were prepared to presume normality at the level of the individual replicate data directly derived from the standard curve, we were less prepared to extend this assumption of approximate normality to the more sparse, more severely skewed summary data items not directly derived from the standard curves, opting for the more conservative non-parametric approach. As such, comparisons of fold change MFI for acetylation, CA-US RNA, fold change in CA-US RNA, HIV DNA, SCA, activation and differentiation markers, integrated DNA, TILDA and ICS between baseline and subsequent time points across all patients used a non-parametric Wilcoxon signed rank test. For each statistical test, a sensitivity analysis was run consisting of parallel non-parametric testing where parametric analysis was chosen, and conversely parallel parametric testing where non-parametric methodologies were used. In each analysis there was no difference in the pattern of significance or, with regards to the modeling, the direction of the coefficients.

Comparisons in CA-US HIV RNA, HIV DNA, SCA, integrated DNA and ICS between pre-vorinostat, on vorinsotat and off vorinostat time periods were also performed using a Generalised Estimating Equations (GEE), using a Gaussian family structure, a link identity function and an exchangeable within-group correlation structure. A robust variance estimator was used secondary to the small sample size and the deviations from normality exhibited in the summary measure data. We further extended the GEE modeling to estimate fixed effects for assay to correct for intra-assay variability by using approximations proposed by Sutradhar and Rao [61] for GEE and further developed by Feddag et al [62]. Outcome variables were log_10_ transformed. All reported p values were two-tailed. A Bonferroni deflation of significance was applied for multiple comparisons, otherwise p<0.05 was considered significant. All analyses were conducted in Stata version 12 (StataCorp, College Station, Texas).

## Supporting Information

Figure S1 **Changes in latently infected cells.** Latently infected cells were quantified in purified total CD4+ T-cells by measuring (A) integrated HIV DNA per million CD4+ T-cells and (B) inducible virus by the Tat Rev Inducible Limiting Dilution Assay (TILDA) as CA-MS RNA positive cells per million CD4+ T-cells.
(EPS)

Figure S2 **Fold changes in CA-US RNA.** Fold changes in CA-US HIV RNA in CD4+ T-cells from blood is shown for each study participant in a separate graph. The mean of 4 replicates for each time point was used to calculate fold change above the baseline mean.
(EPS)

Figure S3 **Expression of activation markers on CD4 and CD8 T-cells from blood and rectal tissue.** Flow cytometry was used to quantify CD4 and CD8+ T-cells (upper row) and expression of markers of T-cell activation in CD4+ and CD8+ T-cells from blood (middle three rows) and rectal tissue (bottom row). Black lines indicate change in activation markers of the patient who had a viral rebound (shown in **Figure 4**). Grey shaded box represents the time on vorinostat.
(EPS)

Figure S4 **Vorinostat induced a dynamic and prolonged change in host gene expression evident within two hours.** Gene expression heatmaps of DEG using matched donor supervised analysis (n = 9) comparing baseline to multiple time points collected during the study period including at (A) two hours, (B) eight hours, (C) day one, (D) day 14 and (E) day 84. The top 50 genes were selected as differentially expressed with p<0.05. Red and blue correspond to up- and down-regulated genes respectively.
(EPS)

Figure S5 **Gene and pathway changes following the first and seventh doses of vorinostat.** Venn diagrams show shared (A) gene expression and (B) pathways between matched donor paired samples two hours after the first dose (left hand circle) and two hours after the 7th daily dose of vorinostat (right hand circle). (C) Radial plot (Nakaya modules) illustrating selective enrichment of gene expression in PBMC cell subsets of the day 0+2 hour (red) and day 7+2 hour (green) timepoints. The radial plot shows a wedge of color that points outward (increased expression) or inward (decreased expression). Genesets induced in a specific subset were significantly enriched (adjusted p-value<0.05 denoted by *) among genes upregulated or downregulated with respect to the enrichment score (NES) between groups. All subsets with the exception of myeloid dendritic cells (MDC) were enriched in the same direction between day 0+2 hour and day 7+2 hour timepoint. pDC = plasmacytoid dendritic cells; NK = natural killer cells.
(TIF)

Table S1 **Adverse events possibly, probably or definitely related to vorinostat.**
(DOCX)

Table S2 **Adverse events unrelated to vorinostat.**
(DOCX)

Table S3 **Plasma trough concentrations of antiretroviral agents.**
(DOCX)

Table S4 **Components of the Super Elongation Complex (SEC) are upregulated two hours following vorinostat treatment.**
(DOCX)

Dataset S1 **Complete gene set enrichment lists.**
(ZIP)
