# Supplementary figures and images for: Activation of HIV Transcription with Short-Course Vorinostat in HIV-Infected Patients on Suppressive Antiretroviral Therapy
Source: PLoS Pathog. 2014 Nov 13;10(11):e1004473. doi: 10.1371/journal.ppat.1004473 (PMC4231123; doi:10.1371/journal.ppat.1004473)

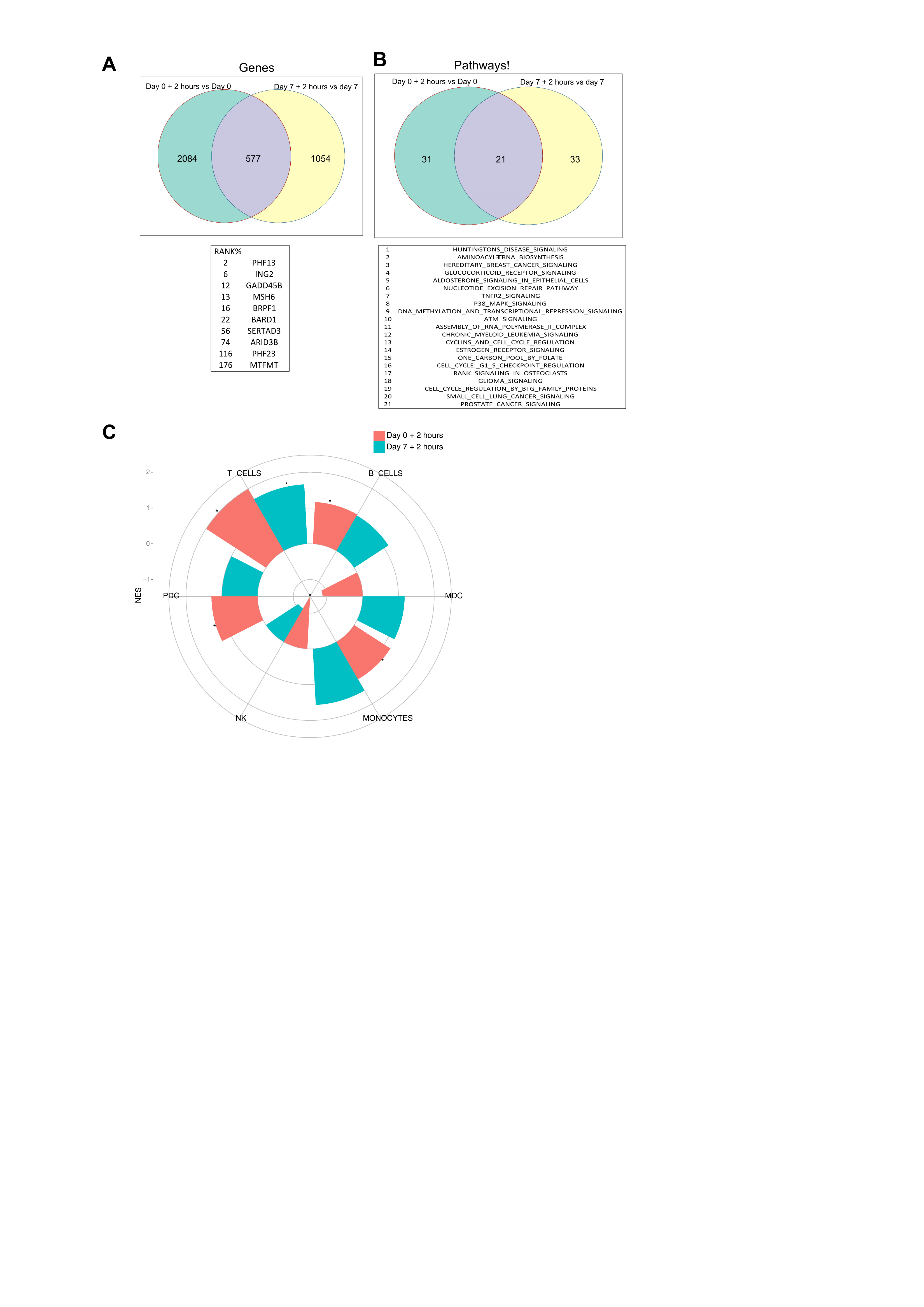

Supplement: Figure S5 — Gene and pathway changes following the first and seventh doses of vorinostat. Venn diagrams show shared (A) gene expression and (B) pathways between matched donor paired samples two hours after the first dose (left hand circle) and two hours after the 7th daily dose of vorinostat (right hand circle). (C) Radial plot (Nakaya modules) illustrating selective enrichment of gene expression in PBMC cell subsets of the day 0+2 hour (red) and day 7+2 hour (green) timepoints. The radial plot shows a wedge of color that points outward (increased expression) or inward (decreased expression). Genesets induced in a specific subset were significantly enriched (adjusted p-value<0.05 denoted by *) among genes upregulated or downregulated with respect to the enrichment score (NES) between groups. All subsets with the exception of myeloid dendritic cells (MDC) were enriched in the same direction between day 0+2 hour and day 7+2 hour timepoint. pDC = plasmacytoid dendritic cells; NK = natural killer cells. (TIF) [file ppat.1004473.s005.tif]

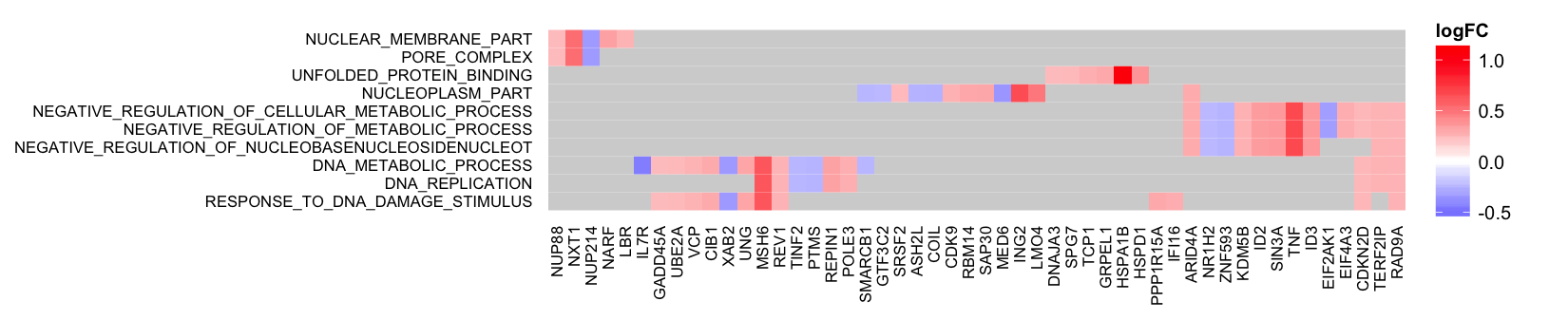

Supplement: Dataset S1 — Complete gene set enrichment lists. (ZIP) [file ppat.1004473.s010.zip › 2hr-BL checkerboard/gsea_Class_On_Vorinostat_Hr_2vsNone_Baseline_0hr.c5.checkerboard.png]

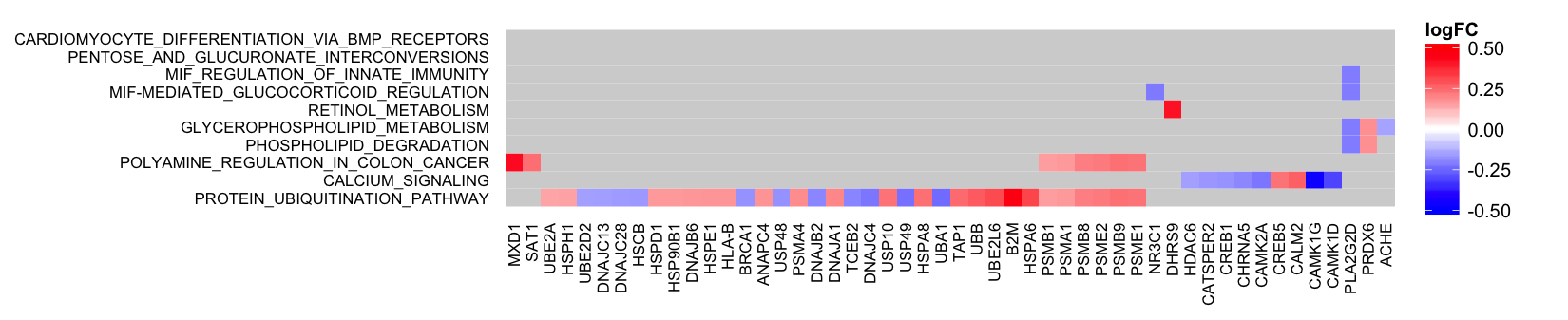

Supplement: Dataset S1 — Complete gene set enrichment lists. (ZIP) [file ppat.1004473.s010.zip › Day84 vs BL checkerboard/gsea_Class_Off_Vorinostat_Day_84vsNone_Baseline_0hr.IPA_CP_R.checkerboard.png]

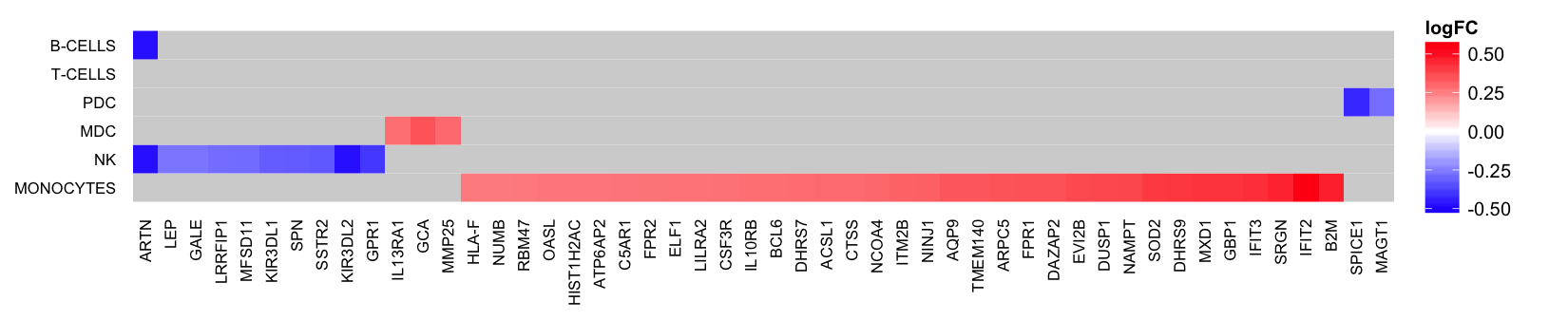

Supplement: Dataset S1 — Complete gene set enrichment lists. (ZIP) [file ppat.1004473.s010.zip › NakayaCheckerboards/gsea_Class_Off_Vorinostat_Day_84vsNone_Baseline_0hr.NakayaHI_2011_NatImmunol.checkerboard.png]

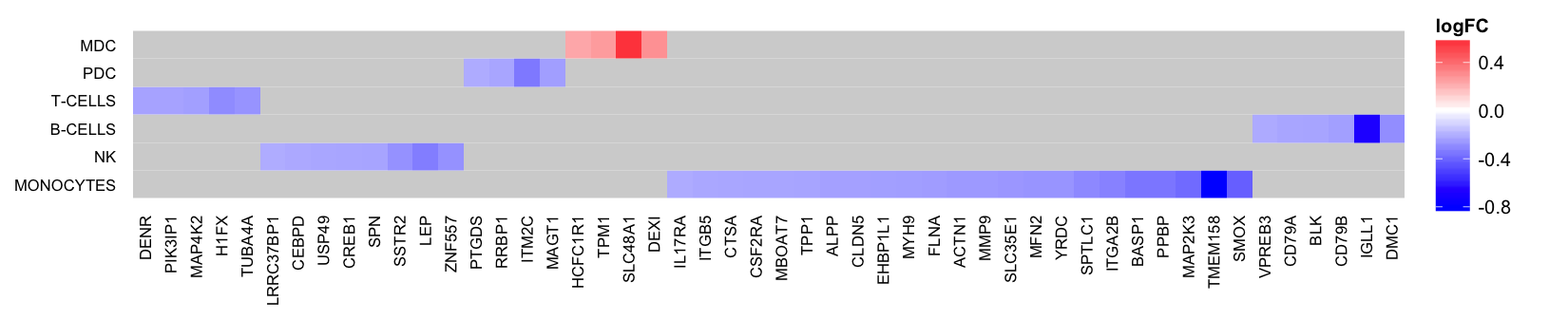

Supplement: Dataset S1 — Complete gene set enrichment lists. (ZIP) [file ppat.1004473.s010.zip › NakayaCheckerboards/gsea_Class_On_Vorinostat_Day_14vsNone_Baseline_0hr.NakayaHI_2011_NatImmunol.checkerboard.png]

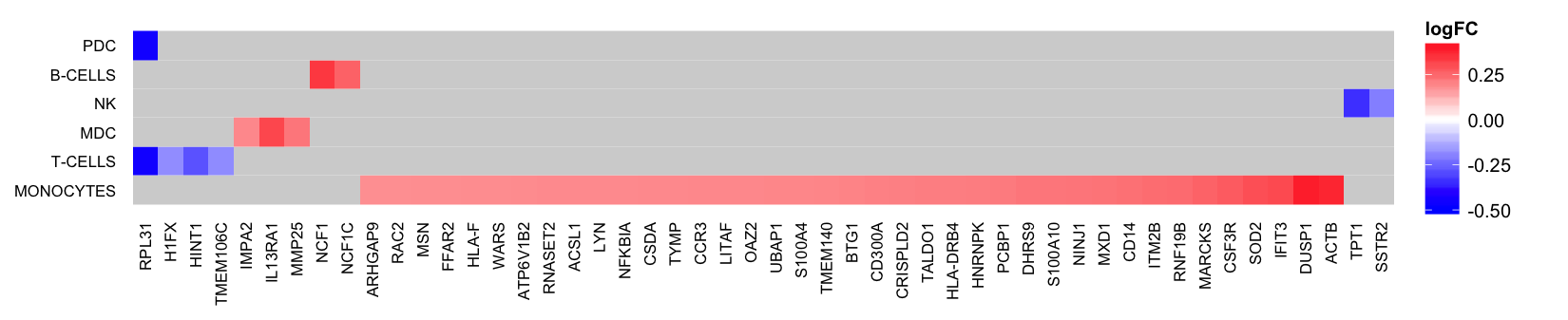

Supplement: Dataset S1 — Complete gene set enrichment lists. (ZIP) [file ppat.1004473.s010.zip › NakayaCheckerboards/gsea_Class_On_Vorinostat_Day_1vsNone_Baseline_0hr.NakayaHI_2011_NatImmunol.checkerboard.png]

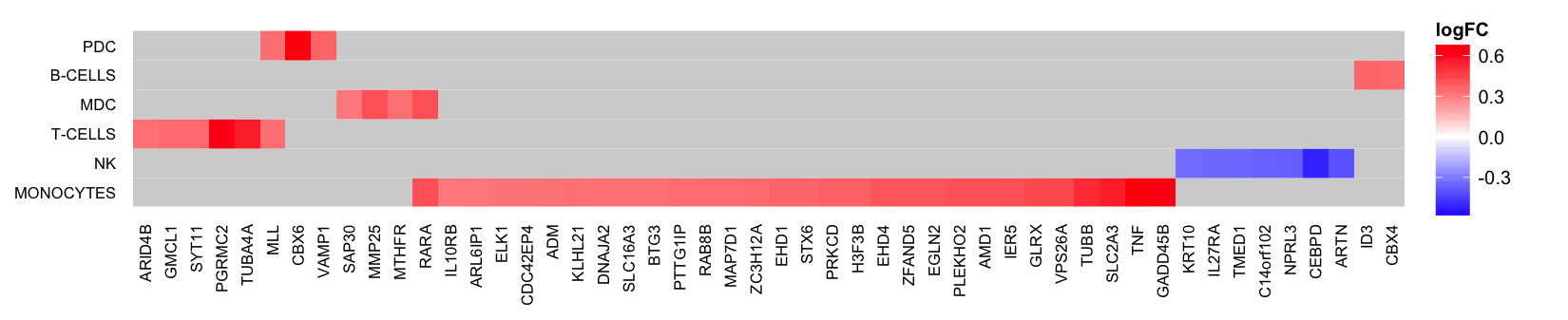

Supplement: Dataset S1 — Complete gene set enrichment lists. (ZIP) [file ppat.1004473.s010.zip › NakayaCheckerboards/gsea_Class_On_Vorinostat_Hr_2vsNone_Baseline_0hr.NakayaHI_2011_NatImmunol.checkerboard.png]

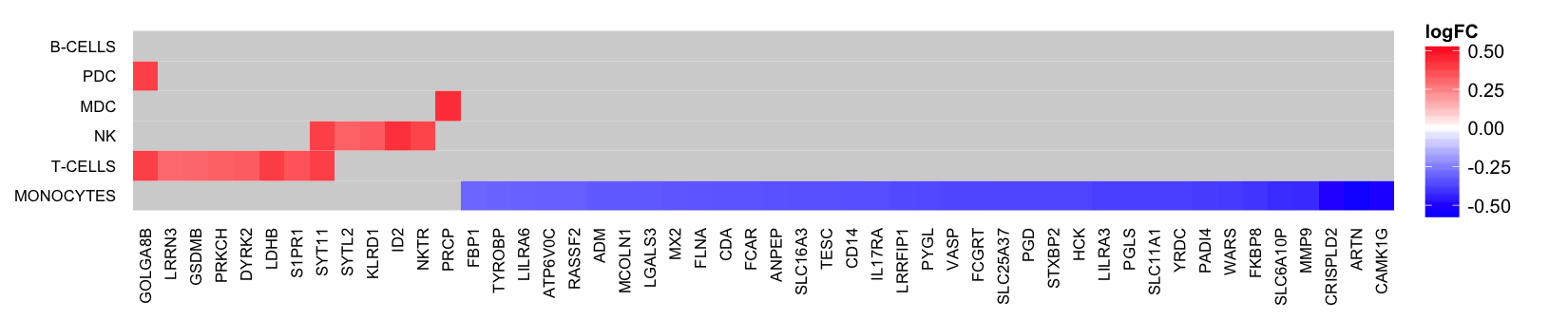

Supplement: Dataset S1 — Complete gene set enrichment lists. (ZIP) [file ppat.1004473.s010.zip › NakayaCheckerboards/gsea_Class_On_Vorinostat_Hr_8vsNone_Baseline_0hr.NakayaHI_2011_NatImmunol.checkerboard.png]
