# Supplementary material for: Activation of HIV Transcription with Short-Course Vorinostat in HIV-Infected Patients on Suppressive Antiretroviral Therapy
Source: PLoS Pathog. 2014 Nov 13;10(11):e1004473. doi: 10.1371/journal.ppat.1004473 (PMC4231123; doi:10.1371/journal.ppat.1004473)
Supplement: Table S1 — Adverse events possibly, probably or definitely related to vorinostat. (DOCX) [file ppat.1004473.s006.docx]

**Table S1: Adverse events possibly, probably or definitely related to vorinostat**

|  | **Adverse Events** | **Grade^a^** | | **Total** | **Day of Onset^b^**  **(Median and Range)** | **Duration of symptoms (Median and Range)** |
| --- | --- | --- | --- | --- | --- | --- |
|  |  | **1** | **2** |  |  |  |
| **Clinical** |  |  |  |  |  |  |
|  | **Gastrointestinal** |  |  |  |  |  |
|  | Diarrhea | 7 | 1 | 8 | 1 (0-7) | 1.5 (1-14) |
|  | Constipation | 2 | - | 2 | 0.5 (0-1) | 20 (15-25) |
|  | Nausea / Vomiting | 3 | 1 | 4 | 1.5 (0-11) | 4 (1-16), |
|  | Dysgeusia | 5 | - | 5 | 0 (0-1) | 10 (1-17) |
|  | Dry Mouth | 3 | - | 3 | 3 (1-6) | 10 (1-23) |
|  | Oral Ulcer | 1 | - | 1 | 3 | 23 |
|  | Flatulence | 1 | - | 1 | 1 | 4 |
|  | Gatroesophegeal Reflux | - | 1 | 1 | 2 | Ongoing at day 84 |
|  | Anorexia | 1 | - | 1 | 6 | 6 |
|  | Gastrointestinal – Other^c^ | 1 | - | 1 | 0 | 1 |
|  | **Neurological** |  |  |  |  |  |
|  | Headache | 3 | 1 | 4 | 0 (0-1) | 9.5 (5-16) |
|  | Impaired Concentration | 3 | 1 | 4 | 0 (0-4) | 5 (1-17) |
|  | Dizziness | 2 | - | 2 | 2 (0-4) | 1 (1-1) |
|  | Dysesthesia | 2 | - | 1 | 0.5 (0-1) | 9.5 (6-13) |
|  | **Constitutional** |  |  |  |  |  |
|  | Lethargy | 4 | 4 | 8 | 0 (0-8) | 7 (1-13) |
|  | Hot flashes | 2 | - | 2 | 0 (0-0) | 7.5 (1-14) |
|  | Myalgia | 2 | - | 2 | 6 (0-12) | 1 (1-1) |
|  | Insomnia | 1 | - | 1 | 0 | 3 |
| **Laboratory** |  |  |  |  |  |  |
|  | Thrombocytopenia | 8 | - | 8 | 14 (14-14) |  |
|  | Neutropenia | 1 |  | 1 | 3 |  |
|  | Hyponatremia | 1 | - | 1 | 7 |  |
|  | Acute Kidney Injury | 1 | - | 1 | 14 |  |
|  | Increased bilirubin | - | 2 | 2 | 7.5 (1-14) |  |
|  | Increased gamma glutamyltransferase | 2 | - | 2 | 4 (1-7) |  |
|  | Increased alkaline phosphatase | 2 | - | 2 | 10.5 (7-14) |  |
|  | Increased creatinine phosphokinase | 1 | - | 1 | 14 |  |

^a^ Grading according to National Cancer Institute Common Terminology Criteria for Adverse Events v 4.0.

^b^ Day when symptom or abnormal laboratory value was first reported or detected. Day 0 is the baseline visit and the first day of drug dosing

^c^ Belching

All adverse events resolved by study end, apart from one participant with worsening of pre-existing gastro-oesophageal reflux symptoms.
