# Supplementary material for: Activation of HIV Transcription with Short-Course Vorinostat in HIV-Infected Patients on Suppressive Antiretroviral Therapy
Source: PLoS Pathog. 2014 Nov 13;10(11):e1004473. doi: 10.1371/journal.ppat.1004473 (PMC4231123; doi:10.1371/journal.ppat.1004473)
Supplement: Table S2 — Adverse events unrelated to vorinostat. (DOCX) [file ppat.1004473.s007.docx]

**Table S2: Adverse events unrelated to vorinostat**

|  | **Grade^a^** | | | |
| --- | --- | --- | --- | --- |
| **Adverse Events** | **1** | **2** | **3** | **Total** |
|  |  |  |  |  |
| **Clinical** |  |  |  |  |
| Hospitalisation |  |  | 1^b^ | 1 |
| Viral syndrome |  |  | 1^c^ | 1 |
| Diarrhea | 3 |  |  | 1 |
| Nausea / Vomiting | 1 |  |  | 1 |
| Dry Mouth | 2 | - | - | 2 |
| Rectal pain | 1 | - | - | 1 |
| Anal lump | 1 |  |  | 1 |
| Oral thrush | 1 |  |  | 1 |
| Headache | 4 | 1 | - | 5 |
| Sore throat | 4 | - | - | 5 |
| Arthralgia | 2 | 1 | - | 3 |
| Arthritis | 1 | - | - | 1 |
| Dyspnea | 2 | - | - | 2 |
| Rhinitis | 1 | 1 | - | 1 |
| Conjunctivitis | 1 | - | - | 1 |
| Palpitations / Chest tightness | - | 1 | - | 1 |
| Cough | 2 |  |  | 2 |
| Upper Respiratory Tract Infection | 2 | - | - | 2 |
| Insomnia | 1 | - | - | 1 |
| Penile Thrush | 1 |  |  | 1 |
|  |  |  |  |  |
| **Laboratory** |  |  |  |  |
| Thrombocytopenia | 1 | - | - | 1 |
| Leucopenia | 2 |  |  | 1 |
| Neutropenia |  | 1 |  | 1 |
| Hypernatremia | 1 | - | - | 1 |
| Hypophosphatemia | - | - | 1^d^ | 1 |
| Increased bilirubin |  | - | 2^d^ | 1 |
| Increased alanine aminotransferase | 3 | 1 | - | 4 |
| Increased gamma glutamyltransferase | 6 | 2 | - | 8 |
| Increased alkaline phosphatase | 2 | - | - | 2 |

^a^ Grading according to National Cancer Institute Common Terminology Criteria for Adverse Events v 4.0.

^b^ The subject was admitted to the Alfred Hospital from Day 27 to Day 29. Onset of illness on Day 25 and all symptoms resolved by Day 34. Illness characterized by abrupt onset of sore neck, sore throat, headache and symptomatic fevers. Pharyngitis on examination. Investigations showed elevated C-reactive protein of 210, normal CT scan of the neck and normal lumbar puncture. Throat swab taken prior to antibiotics revealed no pathogens. Rapid improvement in all symptoms with oral amoxicillin. Presumptive diagnosis of streptococcal pharyngitis.

^c^ The subject reported one day of symptomatic fevers, along with headache, sore throat, diarrhea, arthralgia and a maculopapular rash that involved the upper body including the upper limbs. The subject was in contact with colleagues with a similar clinical illness. Investigations revealed no specific diagnosis, the platelet count was noted to be low at 131 x 10^9^/L. The illness resolved by Day 36 and was thought to be viral in nature

^d^ Present at baseline
