# Supplementary material for: Activation of HIV Transcription with Short-Course Vorinostat in HIV-Infected Patients on Suppressive Antiretroviral Therapy
Source: PLoS Pathog. 2014 Nov 13;10(11):e1004473. doi: 10.1371/journal.ppat.1004473 (PMC4231123; doi:10.1371/journal.ppat.1004473)
Supplement: Table S3 — Plasma trough concentrations of antiretroviral agents. (DOCX) [file ppat.1004473.s008.docx]

**Table S3: Plasma trough concentrations of antiretroviral agents**

| **NNRTI or PI and usual dose timing** | **Baseline**  **(μg/l)** | **Day 14**  **(μg/l)** | **Recommended target trough concentration**  **(μg/l)** |
| --- | --- | --- | --- |
| EFV nightly | 1978 | 2118 | > 1000^a^ |
| NVP twice daily | 4778 | 4647 | > 3000 |
| EFV nightly | 1403 | 1565 | > 1000^a^ |
| NVP twice daily | 4966 | 5165 | > 3000 |
| EFV nightly | 3627 | 3180 | > 1000^a^ |
| EFV nightly | 3302 | 2937 | > 1000^a^ |
| NVP twice daily | 7132 | 4499 | > 3000 |
| NVP twice daily | 5485 | 6681 | > 3000 |
| EFV nightly | 1792 | 1466 | > 1000^a^ |
| DRV once daily | 2634 | 1210 | > 2000 |
| EFV nightly | 6423 | 7164 | > 1000^a^ |
| EFV nightly | 2515 | NR | > 1000^a^ |
| LPV twice daily | 3823 | 8906 | > 2000 |
| NVP once daily | 3129 | 6130 | > 3000 |
| ATV once daily | 2082 | 2371 | > 150 |
| EFV nightly | 2606 | 2071 | > 1000^a^ |
| DRV twice daily | 4377 | 7064 | > 2000 |
| ATV once daily | NR | 1714 | > 150 |
| LPV twice daily | < 100 | 10028 | > 2000 |
| NVP SR once daily | NR | 2407^a^ | > 3000 |

Participants taking cART in the morning withheld the dose and had levels performed in the morning (i.e. a 24 hour trough level). Participants taking cART in the evening did not withhold the dose and had levels performed in the morning (i.e. a 12 hour trough level).

^a^ Recommended target 12 hour trough level

^b^ Performed on Day 21

NOTE: EFV, Efavirenz; NVP, Nevirapine; DRV, Darunavir; LPV, Lopinavir; RAL, Raltegravir; ATV, Atazanavir; NR, no result
