# Supplementary material for: Activation of HIV Transcription with Short-Course Vorinostat in HIV-Infected Patients on Suppressive Antiretroviral Therapy
Source: PLoS Pathog. 2014 Nov 13;10(11):e1004473. doi: 10.1371/journal.ppat.1004473 (PMC4231123; doi:10.1371/journal.ppat.1004473)
Supplement: Table S4 — Components of the Super Elongation Complex (SEC) are upregulated two hours following vorinostat treatment. (DOCX) [file ppat.1004473.s009.docx]

**Table S4: Components of the Super Elongation Complex (SEC) are upregulated two hours following vorinostat treatment.**

| **2 hour vs Baseline** | | | | | | | |
| --- | --- | --- | --- | --- | --- | --- | --- |
| SYMBOL | logFC | AveExpr | T | P.Value | Adj.P.Val | B | FC |
| CCNT2 | 0.07321621 | -0.0033539 | 3.02513297 | 0.00335242 | 0.00335242 | -1.8616253 | 1.05205943 |
| CDK9 | 0.26982784 | -0.0241409 | 4.35031073 | 4.02E-05 | 4.02E-05 | 1.99871785 | 1.20566394 |
| HEXIM1 | -0.1061541 | 0.00205603 | -2.2626057 | 0.02640592 | 0.02640592 | -3.5909389 | -1.0763551 |
| BRD2 | 0.18291599 | 0.0015139 | 2.73210486 | 0.00776063 | 0.00776063 | -2.5746045 | 1.135176 |
| AFF4 | 0.11402365 | -0.0249778 | 2.67049033 | 0.00919241 | 0.00919241 | -2.717056 | 1.08224239 |
| ALL | 0.23540184 | -0.0977871 | 3.00073828 | 0.00360266 | 0.00360266 | -1.9231634 | 1.1772346 |
| **D7+2hours vs D7** | | | | | | | |
| SYMBOL | logFC | AveExpr | T | P.Value | Adj.P.Val | B | FC |
| ELL | 0.2720879 | 0.0234561 | 2.73490757 | 0.00769245 | 0.00769245 | -2.5010902 | 1.20755416 |
| NCOAA3 | 0.20701827 | -0.0150947 | 2.00090565 | 0.04881829 | 0.04881829 | -3.9632851 | 1.15430003 |
| SUPT4H1(DSIF) | 0.29385994 | 0.05747477 | 2.62077873 | 0.01050866 | 0.01050866 | -2.753449 | 1.22591584 |

FC = fold change
